# Design, Synthesis, Spectroscopic Characterisation and In Vitro Cytostatic Evaluation of Novel Bis(coumarin-1,2,3-triazolyl)benzenes and Hybrid Coumarin-1,2,3-triazolyl-aryl Derivatives

**DOI:** 10.3390/molecules27030637

**Published:** 2022-01-19

**Authors:** Kristina Pršir, Ema Horak, Marijeta Kralj, Lidija Uzelac, Sandra Liekens, Ivana Murković Steinberg, Svjetlana Krištafor

**Affiliations:** 1 Department of General and Inorganic Chemistry, Faculty of Chemical Engineering and Technology, University of Zagreb, Marulićev trg 19, 10000 Zagreb, Croatia; kbobanov@fkit.hr (K.P.); Ema.Horak@fidelta.eu (E.H.); imurkov@fkit.hr (I.M.S.); 2 Fidelta Ltd., Prilaz baruna Filipovića 29, 10000 Zagreb, Croatia; 3 Division of Molecular Medicine, Ruđer Bošković Institute, Bijenička cesta 54, 10000 Zagreb, Croatia; Marijeta.Kralj@irb.hr (M.K.); Lidija.Uzelac@irb.hr (L.U.); 4 Laboratory of Virology and Chemotherapy, Department of Microbiology and Immunology, Rega Institute for Medical Research, KU Leuven, 3000 Leuven, Belgium; Sandra.liekens@kuleuven.be

**Keywords:** coumarin, 1,2,3-triazole, benzazole, organic synthesis, cytostatic evaluation, fluorescence

## Abstract

In this work, a series of novel 1,2,3-triazolyl-coumarin hybrid systems were designed as potential antitumour agents. The structural modification of the coumarin ring was carried out by Cu(I)-catalysed Huisgen 1,3-dipolar cycloaddition of 7-azido-4-methylcoumarin and terminal aromatic alkynes to obtain 1,4-disubstituted 1,2,3-triazolyl-coumarin conjugates **2a**–**g**, bis(1,2,3-triazolyl-coumarin)benzenes **2h**–**i** and coumarin-1,2,3-triazolyl-benzazole hybrids **4a**–**b**. The newly synthesised hybrid molecules were investigated for in vitro antitumour activity against five human cancer cell lines, colon carcinoma HCT116, breast carcinoma MCF-7, lung carcinoma H 460, human T-lymphocyte cells CEM, cervix carcinoma cells HeLa, as well as human dermal microvascular endothelial cells (HMEC-1). Most of these compounds showed moderate to pronounced cytotoxic activity, especially towards MCF-7 cell lines with IC_50_ = 0.3–32 μM. In addition, compounds **2a**–**i** and **4a**–**b** were studied by UV-Vis absorption and fluorescence spectroscopy and their basic photophysical parameters were determined.

## 1. Introduction

Coumarin (2H-chromen-2-one) has become a privileged heterocyclic moiety in biomedical chemistry and novel drug discovery. The chemistry of coumarin has been profoundly examined as a result of its wide distribution in nature, and the extensive biological activity of its derivatives, including anticancer [1,2], antioxidant [3], antiviral [4], antibacterial and antitubercular properties [5]. Due to its lipophilic, aromatic and planar structure, the coumarin ring can easily bind different biological targets by π-π, hydrogen or dipole–dipole interactions and therefore is incorporated in the structure of various important bioactive compounds [6]. 

In addition, the therapeutic potential of coumarins strongly depends on their basic structure [7] as seen in simple, bis- and fused polycyclic coumarins [8,9,10,11,12]. Furthermore, the nature and position of substituents on the benzopyrone core can affect the pharmacological properties and biological activities of the resulting molecule. Design and synthesis of coumarin-based hybrid molecules by introducing one or more heterocyclic moieties in the coumarin core is a promising approach in the development of novel therapeutic agents with improved and diverse activity [13]. 

In the context of easy structural modification, the concept of click chemistry has received great attention. The Cu(I)-catalysed Huisgen 1,3-dipolar cycloaddition is considered to be the typical click reaction in which 1,4-disupstituted 1,2,3-triazole is formed [14,15,16]. Over the past decade, the 1,2,3-triazole ring has emerged as one of the most important building blocks in the synthesis of molecular hybrids, and a vast number of molecules have been developed on the basis of this structure [17]. Besides the synthetic simplicity and modular nature of the reaction, aromatic 1,2,3-triazoles obtained in this way have an important effect on the pharmacological profile of the resulting molecule. Triazole rings mostly act as linkers between various heterocyclic motifs in molecular hybrids, but can also directly participate in the binding of the target analyte. Because of the large dipole moment, triazoles can act as hydrogen bond donors, mimicking amide functional group. More importantly, they are resistant to metabolic degradation and hydrolysis, and are stable under acidic, basic and redox conditions in living biological systems. 

**Figure 1:**
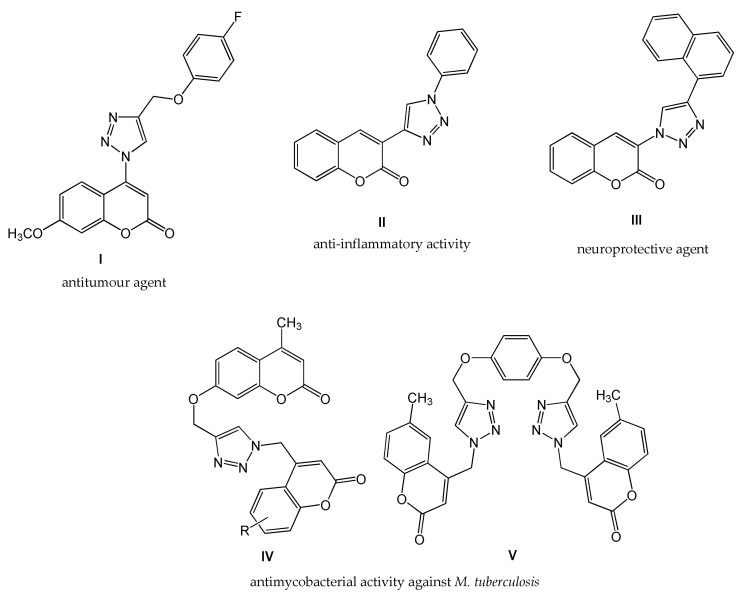
1,2,3-Triazolyl-coumarin-hybrids as bioactive molecules.

In recent years, derivatives conjugated with 1,2,3-triazole at the C-3, C-4 and C-7 positions of the coumarin ring have been evaluated as promising antitumour (**I**) [18,19], anti-inflammatory (**II**) [20], neuroprotective (**III**) [21] and antitubercular agents (**IV**–**V**) [8,22], and representatives are shown in Figure 1. 

**Figure 2:**
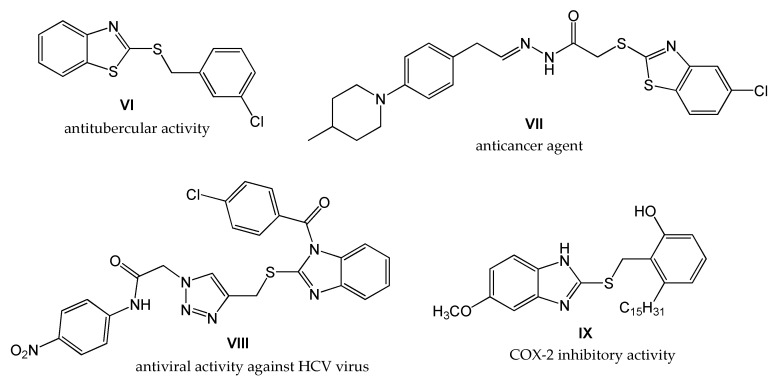
2-Thiobenzazole scaffold in bioactive molecules.

Another essential class of nitrogen containing heterocycles in novel drug development is benzofused azoles. Benzimidazole and benzothiazole represent important pharmacophores in medicinal chemistry, primarily because of their structural similarity with naturally occurring molecules. Among the various benzazole-based compounds, 2-thiobenzazoles have attracted considerable attention in medicinal research and their derivatives have been reported as potential antitubercular (**VI**) [23], antiproliferative (**VII**) [24], antiviral (**VIII**) [25] and anti-inflammatory agents (**IX**) [26] (Figure 2).

Despite the large number of known bioactive coumarin-based molecules, there is a need for novel therapeutic agents with improved activity, especially those substituted at C-7 position of the coumarin ring. Taking into account the expressed activity of these three classes of heterocycles, their combination in one target molecule may lead to enhanced bioactivity when compared to individual entities. This type of molecular hybridisation is considered an effective and powerful tool in drug discovery for development of novel hybrids or conjugates with improved and diverse pharmacological properties [13,17]. Therefore, we have employed a molecular hybridisation strategy in order to design new hybrids that incorporate coumarin, 1,2,3-triazole and/or benzofused benzazole moieties in their scaffold. Here, we report the synthesis of hybrids containing coumarin and 1,2,3-triazole moieties in one scaffold, via a Cu(I)-catalysed 1,3-dipolar cycloaddition reaction of 7-azidocoumarin and terminal alkynes. The compounds consist of 7-(1,2,3-triazolyl)coumarin as a central backbone and aryl-substituted subunit linked to the 4-position of the triazole ring. Moreover, planar bis-(coumarin-1,2,3-triazolyl)benzenes were designed by connecting two 7-(1,2,3-triazolyl)coumarin nuclei to enhance the activity allowing additional interactions. Further modification of the triazolyl-coumarin core was carried out by introducing benzofused heterocycles through a thiomethylene linker. In addition to the compounds being potentially biologically active, fluorescence was observed with some of them, making them potentially suitable for application in fluorescence bioimaging. Coumarins are well-known fluorophores in labelling of biomolecules [27] because of the relatively large Stokes shift and high quantum yields, while in combination with 1,2,3-triazoles, new opportunities are opening up to investigate their potential as fluorescent probes for detecting biologically and environmentally important metal cations [28,29], anions [30] and biomolecules [31]. Therefore, the antiproliferative activity and basic photophysical properties of these newly prepared hybrids has been examined. 

## 2. Results and Discussion

### 2.1. Synthesis

**Scheme 1:**
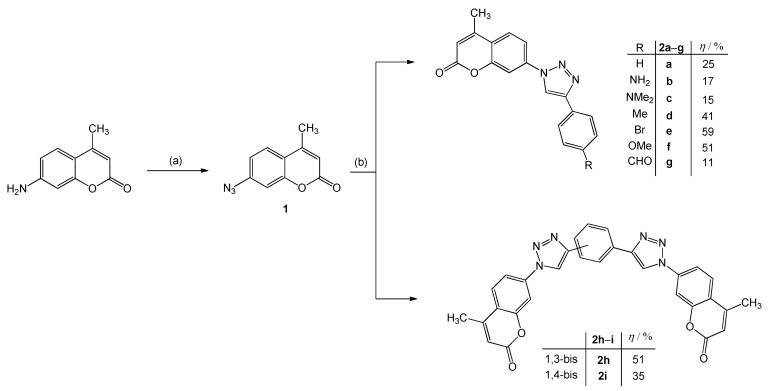
Synthesis of triazolyl-coumarin conjugates **2**. Reagents and conditions: (a) Method A: t-BuONO, TMSN_3_, CH_3_CN, rt., 2 h; Method B: p-TsOH, NaNO_2_, NaN_3_, rt., overnight; (b) alkyne, CuSO_4_, Na ascorbate, TBTA, t-BuOH/H_2_O/CH_2_Cl_2_, 24–72 h, 25–60 °C.

The synthesis of 1,2,3-triazolyl-coumarin hybrids **2a**–**i** is outlined in Scheme 1. Azido coumarin **1**, as a key precursor, was synthesised by two methods using commercially available 7-amino-4-methylcoumarin as starting material. Transformation of amino group to azido substituent in **1** comprised of diazotization and nucleophilic substitution of traditional diazonium salt with azidotrimethylsilane, TMSN_3_ (Method A) [32] or diazotization and tosylation followed by nucleophilic substitution of corresponding coumarinyldiazonium tosylate (ArN_2_^+^TsO^−^) with NaN_3_ as azide source (Method B) [33]. In the simple and practical Method A, tert-butyl nitrite (t-BuONO) was used as diazotizing agent for 7-amino-4-methylcoumarin followed by reaction of arenediazonium salt thus obtained with TMSN_3_. Alternatively, Method B involved a one-pot procedure to azidocoumarin **1** via the non-usual diazonium tosylate as an intermediate. Conversion of 7-amino-4-methylcoumarin to the corresponding azide **1** started by diazotization with NaNO_2_ in water in the presence of p-toluenesulfonic acid, PTSA, followed by azide substitution with NaN_3_. Neither method required purification and afforded the pure azidocoumarin **1** without isolation of diazonium intermediates, but Method B provided **1** in better yield (68% and 40%, respectively). Azidocoumarin **1** was then subjected to copper-catalysed Huisgen 1,3-dipolar cycloaddition with various commercially available or synthesised aromatic terminal alkynes and target 1,4-disubstituted 1,2,3-triazolyl-coumarin conjugates **2a**–**i** were prepared. 

**Scheme 2:**
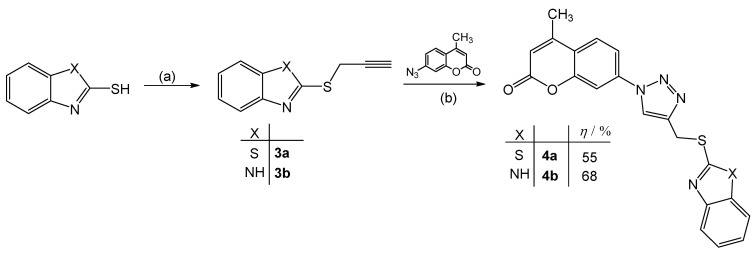
Synthesis of triazolyl-coumarin conjugates **4**. Reagents and conditions: (a) propargyl bromide, K_2_CO_3_, THF, 60 °C, 4 h; (b) CuSO_4_, Na-ascorbate, t-BuOH/H_2_O (1:1), rt., 24 h.

The Cu(I) catalyst was generated in situ from Cu(II) salt using sodium ascorbate as reducing agent. To enhance the catalytic effect of Cu(I) in the azide-alkyne click reaction, polytriazolylamine ligand, tris[(1-benzyl-1H-1,2,3-triazol-4-yl)methyl]amine, TBTA [34] was also used and compounds **2a**–**i** were obtained in low to moderate yields (11% to 59%). Propargylated benzazole derivatives **3a** and **3b** were prepared via nucleophilic addition of propargyl bromide with 2-mercaptobenzthiazole or 2-mercaptobenzmidazole in the presence of K_2_CO_3_ as a base (Scheme 2). Finally, the Cu-catalysed Huisgen cycloaddition reaction of azidocoumarin **1** and propargyl benzazoles **3a** and **3b** yielded the desired coumarin-triazole-benzazole hybrids **4a** and **4b**, respectively.

### 2.2. Antitumoral Activity In Vitro

**Figure 3:**
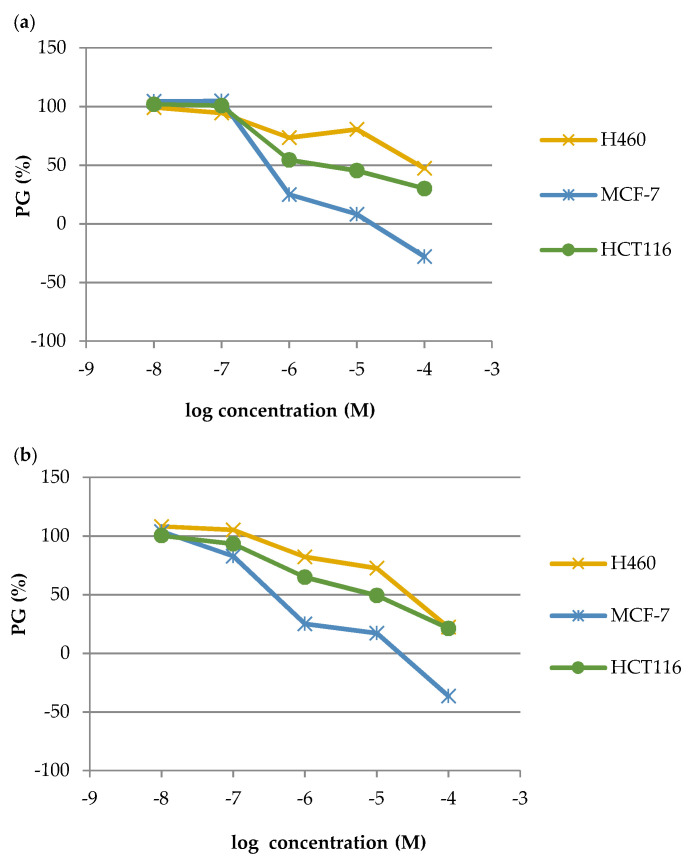
Concentration-response profiles for compounds **2b** (**a**) and **2h** (**b**) tested in vitro on human tumour cell lines HCT116, MCF-7 and H460.

Compounds **2a**–**i**, **4a** and **4b** were evaluated for their in vitro cytostatic activities against a panel of human malignant tumour cell lines, as follows: colon carcinoma (HCT116), breast adenocarcinoma (MCF-7), lung carcinoma (H 460), human T-lymphocyte cells (CEM), human cervix carcinoma cells (HeLa). In addition, we included one non-tumour cell line, human dermal microvascular endothelial cells (HMEC-1), to evaluate the selectivity of the newly prepared compounds. The biological data are presented in Table 1 and Figure 3. Amongst all coumarin-1,2,3-triazole-aryl hybrids **2a**–**g**, compound **2b** bearing an aminophenyl moiety showed the most pronounced cell cytotoxicity and selectivity against the MCF-7 cell line with an IC_50_ = 0.6 μM. Besides, compound **2b** also showed significant cytotoxic activity against HCT116 cell line with IC_50_ = 3 μM. Interestingly, its *N,N*-dimethylated analogue **2c** indicated loss of activity and retained only moderate inhibitory effect against MCF-7 with IC_50_ = 21 μM. Furthermore, compound **2a** with an unsubstituted phenyl moiety at 1,2,3-triazole ring did not show any specific cytostatic activity. Moreover, introduction of methyl (**2d**), methoxy (**2f**) or formyl substituents (**2g**) at the phenyl moiety of the molecular structure did not have any favourable effect on the cytostatic activity of the conjugates, and only moderate activity against HCT116 cell line was observed (IC_50_ = 36, 22 and 77 μM, respectively). In addition, the bromo substituent on the phenyl ring in **2e** had mild influence on cytotoxicity against tested cell lines. 

Bis(coumarin-1,2,3-triazolyl)benzenes **2h** and **2i** were generally the most cytotoxic compounds, especially 1,3-bis(coumarin-triazolyl) derivative **2h** with strong inhibitory activity against breast (MCF-7: IC_50_ = 0.3 μM) and colon carcinoma (HCT116: IC_50_ = 9 μM). Its 1,4-disubstituted structural isomer **2i** displayed slightly reduced cytotoxicity with IC_50_ = 1 and 7 μM against MCF-7 and HCT116 cells, respectively. 

Two coumarin-1,2,3-triazole-benzazole hybrids **4a** and **4b** were also evaluated and demonstrated almost the same moderate activity against all cell lines. Additionally, benzothiazole derivative **4a** showed more pronounced selectivity than its benzimidazole congener **4b** against MCF-7 cell lines, with IC_50_ = 3 μM. 

Interestingly, MCF-7 cell line was especially sensitive towards almost all synthesised compounds with IC_50_ = 0.3–32 μM except for **2d**, **f** and **g**, which were devoid of activity against this cancer cell line. The HMEC-1 cell line was almost completely insensitive to all tested compounds.

### 2.3. Antiviral Activities In Vitro

The antiviral activity of synthesised compounds (**2a**–**c**, **2h**–**i** and **4a**–**b**) was tested against Herpes simplex virus 1 and 2, vaccinia virus, adeno virus-2, human coronavirus, vesicular stomatitis virus, respiratory syncytial virus, reovirus-1, Sindbis virus, Coxsackie virus B4, Punta Toro virus, varicella-zoster virus, cytomegalovirus and yellow fever virus. None of the compounds showed any significant antiviral activity (data not shown).

### 2.4. Spectroscopic Characterisation

The structures of all compounds were determined by NMR analysis (based on inspecting H–H coupling constants and chemical shifts). Generally, ^1^H NMR spectra of all 1,2,3-triazolyl-coumarin derivatives showed characteristic protons at C5 of triazole at 8.95–9.66 ppm, and proton at C2 of coumarin moiety at approximately 6.5 ppm. ^1^H and ^13^C NMR and HRMS spectral data for compounds **2a**–**i** and **4a**–**b** are summarised in Table S1.

#### UV-Visible Absorption and Fluorescence Emission Spectra

**Figure 4:**
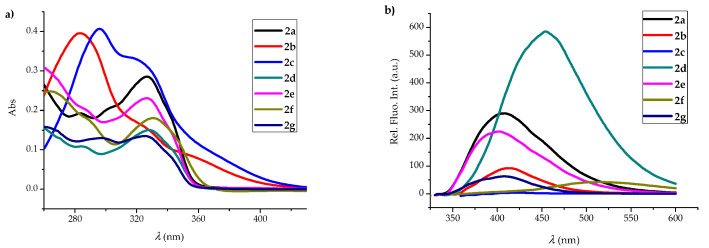

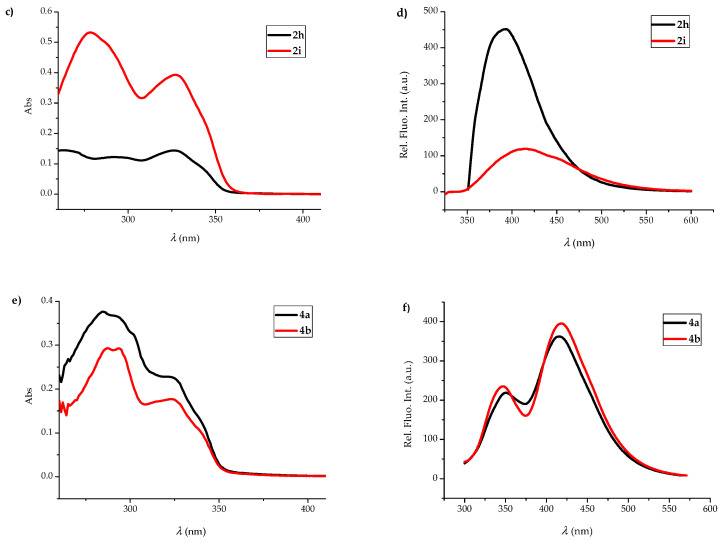
UV-Vis absorption (**a**,**c**,**e**) and fluorescence emission spectra (**b**,**d**,**f**) of: **2a**–**g** (**a**,**b**); **2h**–**2i** (**c**,**d**) and **4a**–**b** (**e**,**f**) in DMSO (*c* = 10 μM), *λ*_exc_ = *λ*_abs,max_.

Coumarin derivatives are widely used as fluorophores in fluorescent probes and fluorescent tags for biological molecules. Conventional coumarin dyes typically absorb at and are excited by UV light, with emission in the blue spectral region [35]. However, excitation at short wavelengths is not desirable for bioimaging applications because of possible autofluorescence and photo-induced damage of tissue. With an appropriate substitution in the 3- and/or 7-position of the coumarin ring, the derivatives obtained can emit strong fluorescence in the blue-green region (400–550 nm). For example, introduction of an 1,2,3-triazole ring at 3- or 7-position of coumarin has been shown to strongly affect their fluorescent properties [36,37]. Therefore, spectroscopic characterisation of newly synthesised coumarin derivatives **2a**–**g** and **4a**–**b** containing different substituents conjugated via 1,2,3-triazole linker, as well as bis(triazolyl-coumarin)benzenes **2h**–**i**, was conducted. UV-Vis absorption and fluorescence emission spectra were measured in dimethyl sulfoxide (DMSO) at the compound concentration of 1.0 × 10^−5^ mol dm^−3^ and are presented in Figure 4.

- UV-Vis absorption spectra

The coumarin oxygen atom has *sp*^2^ hybridization and is a part of the π-system of the molecule, so the spectrum may show an n→π* electronic transition in addition to a number of π→π* transitions. Compound **2a**, containing an unsubstituted phenyl ring exhibited spectra with the absorption maximum at 328 nm. Compounds **2a**–**g** displayed absorption spectra of similar general shape as **2a**, with the main absorption band in the range of 325–331 nm and generally lower molar absorption coefficient depending on substituents on the phenyl ring (*ε* = 13,500–23,200 M^–1^ cm^–1^) (Figure 4a). However, aminophenyl and *N*,*N*-dimethylaminophenyl substituted derivatives **2b** and **2c** show greatly enhanced absorption in the range 284–296 nm with high extinction coefficients (*ε* > 39,000 M^−1^ cm^−1^) accompanied by a shoulder peak at around 325 nm. Methoxyphenyl-substituted **2f** showed slight bathochromic shift (4 to 6 nm) compared to compounds **2a**–**e** and **2g**. The observed absorption bands are assigned to the π→π* electronic transitions characteristic for the whole coumarin ring system, while the expected n→π* transition may be submerged under the stronger π→π* transition.

1,4-Bis(triazolyl-coumarin)benzene **2i** showed the most intense absorption bands at 278 and 326 nm (*ε* = 53,700 and 39,700 M^−1^ cm^−1^) in the series of studied compounds **2**. 1,3-bis-disubstituted analogue **2h** showed slightly different absorption spectra, exhibiting one noticeable maximum at 326 nm and a shoulder at 292 nm with considerably lower molar extinction coefficients (*ε* = 14,400 M^−1^ cm^−1^ and 12,200 M^−1^ cm^−1^) (Figure 4c). 

Introduction of benzazole ring to coumarin-triazolyl hybrids **4a** and **4b** resulted in absorption spectra with two maxima in the range of 285–292 nm (*ε* = 29,200–38,800 M^−1^ cm^−1^) and a shoulder around 325 nm (*ε* = 17,600–22,500 M^−1^ cm^–1^) (Figure 4e).

- Fluorescence emission spectra

Considering the fluorescence emission spectra, compounds **2a**–**g** displayed single emission bands with variable emission maxima and intensity, depending on the substituents at 1,2,3-triazole, as shown in Figure 4b. Phenyl-unsubstituted derivative **2a** displayed an emission band with a maxima at 406 nm, while substitution at phenyl ring in compounds **2b**–**g** resulted in bathochromic shifts of emission maxima (5 to 111 nm), with the exception of bromo-substituted derivative **2e**. As shown in Figure 4b, *p*-tolyl substituted derivative **2d** showed the most pronounced fluorescence intensity at 445 nm, while **2c** did not fluoresce at all in DMSO. Despite emitting at 517 nm and showing the largest Stokes shift of all compounds (187 nm), **2f** displayed quite low fluorescence in comparison to other conjugates. It should be noted that all the derivatives **2** exhibit large Stokes shifts (64–187 nm) and therefore have the potential for application as molecular probes and light-emitting markers in chemistry and biology. Additionally, it can be noticed that introduction of strong electron-donating substituents on the phenyl ring results in lowering of quantum yields. As the electron–donating strength of substituents on the phenyl ring decreases in order **2c** (NMe_2_), **2b** (NH_2_), **2f** (OMe), the respective quantum yields increase in the same order 0.004, 0.035, 0.058 reaching the highest value of 0.445 for weakly electron-donating methyl group in **2d**. The respective molar absorption coefficients follow the same trend and increase with increasing electron-donating ability of the substituent ranging from 15,100 for **2d** to 41,000 M^−1^ cm^−1^ for **2c**.

Bis(coumarin-triazolyl)benzenes showed rather different emission spectra. 1,3-bis derivative **2h** exhibited an emission maximum at the lowest wavelength of all compounds, emitting at 394 nm. However, 1,4-disubstitution on benzene ring resulted in a bathocromic shift, and the observed emission maximum of **2i** was at 415 nm (Figure 4d). 

Interestingly, triazolyl-coumarins with benzazole moiety **4a** and **4b** showed dual emission in DMSO, with maxima at 350 and 416 nm (**4a**), and 357 and 418 nm (**4b**) (Figure 3c). Organic fluorophores with dual emission properties are especially interesting for application in white light electroluminescence and luminescent bioimaging [38]. Dual emission most commonly occurs as a result of photoinduced structural rearrangement and charge re-distribution in fluorophore molecules, so further studies are underway to investigate this. The basic photophysical properties of **2a**–**i** and **4a**–**b** in DMSO are summarised in Table 2. 

## 3. Materials and Methods

### 3.1. General Methods

All chemicals and solvents were purchased from commercial suppliers Acros (Antwerp, Belgium), Sigma Aldrich (St. Louis, MO, USA) or Fluka (Buchs, Switzerland) and used as received without further purification. ^1^H and ^13^C NMR spectra were recorded on a Bruker Avance 300 (Bruker, Billerica, MA, USA) or Bruker Avance 600 (Bruker, Billerica, MA, USA) at 300, 600, and 150 and 75 MHz, respectively. All NMR spectra were measured in DMSO-*d*_6_ solutions, while chemical shifts are reported in ppm (*δ*) relative to TMS as the internal standard. Individual resonances were assigned on the basis of their chemical shifts, signal intensities, multiplicity of resonances. Melting points were recorded on SMP10 Bibby apparatus (Barloworld Scientific, Staffordshire, UK). The electronic absorption spectra were recorded on a Varian Cary 50 spectrometer (Varian, Inc., Palo Alto, California, USA) using a quartz cuvette (1 cm). Mass spectra were recorded on Bruker Microflex MALDI/TOF mass spectrometer (Bruker, Billerica, MA, USA) using positive ionization. All compounds were routinely checked by TLC using Merck silica gel 60F-254 glass plates. The spectral data can be found in Supplementary Materials in Table S1 and Figures S1–S25.

### 3.2. Procedures for the Preparation of Compounds

#### 3.2.1. 7-Azido-4-methyl-2H-chromen-2-one (**1**)

*Method A:* 7-Amino-4-methylcoumarin (300.0 mg, 1.71 mmol) was dissolved in anhydrous acetonitrile (10 mL) and cooled to 0 °C in an ice bath. Then, *t*-BuONO (0.34 mL, 2.86 mmol) and TMSN_3_ (0.28 mL, 2.11 mmol) were added dropwise and the reaction mixture was stirred at room temperature for 2 h. The solvent was evaporated under reduced pressure and crude orange/brown compound **1** (136.0 mg, 40%) was isolated.

*Method B:* To a solution of 7-amino-4-methylcoumarin (100.0 mg, 0.57 mmol) and *p*-toluenesulphonic acid monohydrate (977.4 mg, 5.14 mmol) in 5.0 mL of H_2_O, NaNO_2_ (354.4 mg, 5.14 mmol) was gradually added. After stirring of the reaction mixture for 1 h at room temperature, NaN_3_ (59.4 mg, 0.91 mmol) was added and immediate emission of N_2_ was observed. The resulting mixture was stirred overnight, filtered, washed with water and pure compound **1** (78.0 mg, 68%) as orange/brown powder was isolated; m.p. 163–172 °C. 

^1^H NMR (600 MHz, DMSO-*d*_6_): *δ*/ppm = 7.77 (d, 1H, *J* = 8.5 Hz), 7.14–7.10 (m, 2H), 6.32 (s, 1H), 2.40 (s, 3H). ^13^C NMR (75 MHz, DMSO-*d*_6_): *δ*/ppm = 154.5, 153.4, 143.8, 128.5, 127.5, 117.2, 116.1, 113.7, 107.3, 18.6. 

#### 3.2.2. General Procedure for the Synthesis of Propargylated Benzothiazole (**3a**) and Benzimidazole (**3b**) Derivatives

To a solution of 2-mercaptobenzothiazole (600.0 mg, 3.59 mmol) or 2-mercaptobenzimidazole (600.0 mg, 4.0 mmol) in dry THF (5 mL) triethylamine (1.5 eq) was added and reaction mixture was stirred for 15 min at room temperature. Afterwards, propargyl bromide (1.0 eq) was added and mixture was heated to reflux till its completion. After 4h the solvent was evaporated under reduced pressure and crude residue was purified by recrystallisation from ethanol (5 mL). 

##### 2-(Prop-2-ynylthio)benzo[*d*]thiazole (**3a**)

Brown crystals of compound **3a** were isolated (240.0 mg, 33%); m.p. 95 °C. 

^1^H NMR (300 MHz, DMSO-*d*_6_): *δ*/ppm = 8.05 (d, 1H, *J* = 7.9 Hz), 7.89 (d, 1H, *J* = 7.5 Hz), 7.48 (td, 1H, *J*_1_ = 7.7 Hz, *J*_2_ = 1.3 Hz), 7.39 (td, 1H, *J*_1_ = 7.6 Hz, *J*_2_ = 1.2 Hz), 4.25 (d, 2H, *J* = 2.6 Hz), 3.31 (bs, 1H). ^13^C NMR (75 MHz, DMSO-*d*_6_): *δ*/ppm = 165.5, 153.0, 135.3, 126.9, 125.1, 122.4, 121.8, 79.8, 75.2, 21.5. 

##### 2-(Prop-2-ynylthio)-1H-benzo[*d*]imidazole (**3b**)

White crystals of compound **3b** were isolated (197.0 mg, 26%); m.p. 173 °C.

^1^H NMR (600 MHz, DMSO-*d*_6_): *δ*/ppm = 12.63 (s, 1H), 7.53 (d, 1H, *J* = 7.4 Hz), 7.38 (d, 1H, *J* = 7.3 Hz), 7.14–7.11 (m, 2H), 4.13 (s, 2H), 3.19 (s, 1H). ^13^C NMR (75 MHz, DMSO-*d*_6_): *δ*/ppm = 153.6, 148.8, 122.0, 109.9, 80.5, 74.5, 20.2. 

#### 3.2.3. General Procedure for the Synthesis of Coumarin-Triazolyl-Aryl Conjugates **2a**–**g**

7-Azido-4-methyl-2H-chromen-2-one **1** (30.0 mg, 0.15 mmol) and the corresponding aromatic alkyne (1 eq) were dissolved in *t*-BuOH/H_2_O/CH_2_Cl_2_ = 1:2:1 (8.0 mL). Next, 1 M CuSO_4_ × 5 H_2_O (0.04 mL), Na-ascorbate (33.7 mg, 0.17 mmol) and TBTA (tris[(1-benzyl-1H-1,2,3-triazol-4-yl)methyl]amine) (4.0 mg, 0.0075 mmol) were added to this solution. Reaction mixture was stirred for 48 h at room temperature. Progress of the reaction was monitored by TLC using dichloromethane and methanol as eluent. The reaction mixture was evaporated to dryness under reduced pressure and crude residue was purified by column chromatography on silica gel with dichloromethane/methanol (200:1) as eluent to afford **2a**–**g** in 11%–59% yield. 

##### 4-Methyl-7-(4-phenyl-1H-1,2,3-triazol-1-yl)-2H-chromen-2-one (**2a**)

Compound **2a** was prepared according to above procedure starting from azidocoumarin **1** and ethynylbenzene (1 eq). After stirring for 48 h and purification, compound **2a** (28.0 mg, 25%) was isolated as yellow powder; m.p. 267–270 °C.

^1^H NMR (300 MHz, DMSO-*d*_6_): *δ*/ppm = 9.49 (s, 1H), 8.05 (s, 3H), 7.97 (d, 2H, *J* = 7.1 Hz), 7.56–7.51 (m, 2H), 7.45–7.40 (m, 1H), 6.52 (s, 1H), 2.74 (s, 3H). ^13^C NMR (75 MHz, DMSO-*d*_6_): *δ*/ppm = 159.9, 154.2, 153.2, 148.1, 139.0, 130.4, 129.6, 129.0, 127.8, 125.9, 120.3, 120.0, 115.9, 115.2, 107.8, 18.6. HRMS (MALDI TOF/TOF) *m*/*z* calcd for C_18_H_14_N_3_O_2_ [M + H]^+^: 304.1081, found [M + H]^+^: 304.1096.

##### 7-[4-(4-Aminophenyl)-1H-1,2,3-triazol-1-yl]-4-methyl-2H-chromene-2-one (**2b**)

Compound **2b** was synthesised as described from azidocoumarin **1** and 4-ethynylaniline (1 eq). After stirring for 48 h and column chromatography, dark yellow powder of **2b** was obtained (30.0 mg, 17%); m.p. 269–273 °C. 

^1^H NMR (300 MHz, DMSO-*d*_6_): *δ*/ppm = 9.17 (s, 1H), 8.01 (s, 3H), 7.60 (d, 2H, *J* = 8.4 Hz), 6.67 (d, 2H, *J* = 8.5 Hz), 6.49 (s, 1H), 5.35 (s, 2H) 2.27 (s, 3H). ^13^C NMR (75 MHz, DMSO-*d*_6_): *δ*/ppm = 152.3, 149.7, 149.2, 147.5, 139.2, 135.4, 127.7, 127.0, 119.7, 117.8, 117.7, 115.7, 115.0, 114.4, 107.5, 18.6. HRMS (MALDI TOF/TOF) *m*/*z* calcd for C_18_H_15_N_4_O_2_ [M + H]^+^: 319.1190, found [M + H]^+^: 319.1203. 

##### 7-[4-(4-(Dimethylamino)phenyl]-1H-1,2,3-triazol-1-yl)-4-methyl-2H-chromen-2-one (**2c**)

Compound **2c** was synthesised according to described procedure, from azidocoumarin **1** and 4-ethynyl-*N*,*N*-dimethylaniline (1 eq) after stirring for 48 h. After column chromatography compound **2c** (15.7 mg, 15%) was obtained as pale yellow powder; m.p. 275–279 °C.

^1^H NMR (600 MHz, DMSO-*d*_6_): *δ*/ppm = 9.25 (s, 1H), 8.02 (s, 3H), 7.76 (d, 2H, *J* = 8.5 Hz), 6.84 (d, 2H, *J* = 8.7 Hz), 6.49 (s, 1H), 2.97 (s, 6H), 1.23 (s, 3H). ^13^C NMR (75 MHz, DMSO-*d*_6_): *δ*/ppm = 167.3, 159.9, 153.3, 150.9, 148.8, 128.4, 127.7, 126.8, 125.9, 118.1, 118.0, 115.7, 115.0, 112.8, 107.6, 29.5, 18.6. HRMS (MALDI TOF/TOF) *m*/*z* calcd for C_20_H_19_N_4_O_2_ [M + H]^+^: 347.1503, found [M + H]^+^: 347.1496.

##### 4-Methyl-7-[4-(*p*-tolyl)-1H-1,2,3-triazol-1-yl]-2H-chromen-2-one (**2d**)

Compound **2d** was prepared following above procedure from azidocoumarin **1** and 1-ethynyl-4-methylbenzene (1 eq). The stirring of reaction mixture for 48 h and purification by column chromatography yielded **2d** (50.3 mg, 41%) as pale yellow powder; m.p. 269–272 °C.

^1^H NMR (300 MHz, DMSO-*d*_6_): *δ*/ppm = 9.42 (s, 1H), 8.03 (s, 3H), 7.84 (d, 2H, *J* = 8.1 Hz), 7.33 (d, 2H, *J* = 7.9 Hz), 6.50 (s, 1H), 2.36 (s, 3H), 1.23 (s, 3H). ^13^C NMR (150 MHz, DMSO-*d*_6_): *δ*/ppm = 159.4, 153.7, 152.7, 147.7, 138.5, 137.9, 129.6 (2C), 127.3, 127.1, 125.3 (2C), 119.4, 115.3, 114.6, 112.7, 107.3, 20.8, 18.6. HRMS (MALDI TOF/TOF) *m*/*z* calcd for C_19_H_16_N_3_O_2_ [M + H]^+^: 318.1237, found [M + H]^+^: 318.1239.

##### 7-[4-(4-Bromophenyl)-1H-1,2,3-triazol-1-yl]-4-methyl-2H-chromen-2-one (**2e**)

Compound **2e** was obtained according to above procedure starting from azidocoumarin **1** and 1-bromo-4-ethynylbenzene (1 eq). The reaction mixture was stirred for 48 h and purified to yield compound **2e** (83.0 mg, 59%) as yellow powder; m.p. 280–285 °C.

^1^H NMR (300 MHz, DMSO-*d*_6_): *δ*/ppm = 9.53 (s, 1H), 8.03 (s, 3H), 7.91 (d, 2H, *J* = 8.5 Hz), 7.74 (d, 2H, *J* = 8.5 Hz), 6.51 (s, 1H), 2.73 (s, 3H). ^13^C NMR (75 MHz, DMSO-*d*_6_): *δ*/ppm = 159.9, 154.1, 153.2, 147.1, 138.9, 132.6, 129.7, 127.8, 122.0, 120.7, 120.1, 115.9, 115.2, 107.9, 18.6. HRMS (MALDI TOF/TOF) *m*/*z* calcd for C_18_H_13_BrN_3_O_2_ [M + H]^+^: 382.0186, found [M + H]^+^: 382.0194. 

##### 7-[4-(4-Methoxyphenyl)-1H-1,2,3-triazol-1-yl]-4-methyl-2H-chromen-2-one (**2f**)

Compound **2f** was synthesised as described from azidocoumarin **1** and1-ethynyl-4-methoxybenzene (1 eq). After stirring for 48 h and column chromatography pale yellow powder of compound **2f** was obtained (62.4 mg, 51%); m.p. 266–270 °C.

^1^H NMR (300 MHz, DMSO-*d*_6_): *δ*/ppm = 9.63 (s, 1H), 8.03 (s, 3H), 7.88 (d, 2H, *J* = 8.8 Hz), 7.09 (d, 2H, *J* = 8.8 Hz), 6.50 (s, 1H), 3.82 (s, 3H) 2.73 (s, 3H). ^13^C NMR (75 MHz, DMSO-*d*_6_): *δ*/ppm = 159.9, 159.9, 154.2, 153.2, 148.1, 139.0, 127.8, 127.8, 122.9, 119.9, 119.2, 115.8, 115.1, 115.0, 107.7, 55.7, 18.6. HRMS (MALDI TOF/TOF) *m*/*z* calcd for C_19_H_16_N_3_O_3_ [M + H]^+^: 334.1186, found [M + H]^+^: 334.1200. 

##### 4-[1-(4-Methyl-2-oxo-2H-chromen-7-yl]-1H-1,2,3-triazol-4-yl)benzaldehyde (**2g**)

Compound **2g** was prepared as described from azidocoumarin **1** and 4-ethynylbenzaldehyde (1 eq). The mixture was stirred for 48 h and chromatographed to yield compound **2g** (13.0 mg, 11%) as yellow powder; m.p. 282–287 °C.

^1^H NMR (300 MHz, DMSO-*d*_6_): *δ*/ppm = 10.12 (s, 1H), 9.66 (s, 1H), 8.48 (s, 1H), 8.28 (d, 1H, *J* = 7.7 Hz), 8.05 (s, 3H), 7.97 (d, 1H, *J* = 7.6 Hz), 7.81–7.76 (m, 1H), 6.51 (s, 1H), 2.73 (s, 3H). ^13^C NMR (75 MHz, DMSO-*d*_6_): *δ*/ppm = 193.0, 168.5, 159.4, 154.6, 153.7, 152.7, 143.2, 138.5, 131.1, 131.0, 130.1, 129.8, 127.4, 125.6, 120.6, 115.4, 114.8, 107.4, 18.1. HRMS (MALDI TOF/TOF) *m*/*z* calcd for C_19_H_14_N_3_O_3_ [M + H]^+^: 332.1030, found [M + H]^+^: 332.1024. 

##### 7-{{4-[(Benzo[*d*]thiazol-2-yl)thio]methyl}-1H-1,2,3-triazol-1-yl}-4-methyl-2H-chromen-2-one (**4a**)

Compound **4a** was synthesised according to above procedure using azidocoumarin **1** and propargylated benzothiazole derivative **3a** (1 eq). After stirring of reaction mixture at 60 °C for 24 h and purification of raw material, crude dark yellow compound **4a** (50.0 mg, 55%) was isolated; m.p. 186–192 °C. 

^1^H NMR (300 MHz, DMSO-*d*_6_): *δ*/ppm = 9.00 (s, 1H), 8.03–7.92 (m, 5H), 7.49 (t, 1H, *J* = 7.6 Hz), 7.40–7.35 (m, 1H), 6.46 (s, 1H), 4.82 (s, 2H), 2.47 (s, 3H). ^13^C NMR (150 MHz, DMSO-*d*_6_): *δ*/ppm = 156.5, 159.4, 153.6, 152.7, 152.5, 144.2, 138.3, 134.7, 127.2, 126.4, 124.6, 122.3, 121.8, 121.3, 119.5, 115.5, 114.7, 107.4, 27.2, 18.1. HRMS (MALDI TOF/TOF) *m*/*z* calcd for C_20_H_14_N_4_O_2_S_2_Na [M + Na]^+^: 429.0450, found [M + Na]^+^: 429.0471. 

##### 7-{4-{[(1H-Benzo[*d*]imidazol-2-yl)thio]methyl}-1H-1,2,3-triazol-1-yl}-4-methyl-2H-chromen-2-one (**4b**)

Compound **4b** was prepared as described starting with azidocoumarin **1** and propargylated benzimidazole derivative **3b** (1 eq). After stirring at 60 °C for 24 h and column chromatography yellow powder of compound **4b** (113.0 mg, 68%) was obtained; m.p. 237–244 °C. 

^1^H NMR (600 MHz, DMSO-*d*_6_): *δ*/ppm = 12.91 (s, 1H), 8.95 (s, 1H), 7.95–7.89 (m, 3H), 7.55 (bs, 1H), 7.44 (bs, 1H), 7.15–7.13 (m, 2H), 6.47 (s, 1H), 4.78 (s, 2H) 2.46 (s, 3H). ^13^C NMR (150 MHz, DMSO-*d*_6_): *δ*/ppm = 159.4, 153.4, 152.7, 152.6, 147.6, 138.5, 137.8, 136.5, 127.3, 127.2, 120.1, 119.4, 116.1, 115.5, 115.1, 114.7, 108.3, 107.5, 31.2, 25.6, 18.0. HRMS (MALDI TOF/TOF) *m*/*z* calcd for C_20_H_15_N_5_O_2_SNa [M + Na]^+^: 412.0839, found [M + Na]^+^: 412.0837.

#### 3.2.4. General Procedure for the Preparation of Bis(coumarin-1,2,3-triazolyl)benzenes **2h**–**i**

7-azido-4-methyl-2H-chromen-2-one **1** (30.0 mg, 0.15 mmol) and the corresponding alkyne (0.5 eq) were dissolved in *t*-BuOH/H_2_O/CH_2_Cl_2_ = 1:2:1 (8.0 mL). 1 M CuSO_4_ × 5 H_2_O (0.04 mL), Na-ascorbate (33.7 mg, 0.17 mmol) and 4.0 mg TBTA (tris[(1-benzyl-1H-1,2,3-triazol-4-yl)methyl]amine) (0.0075 mmol, 0.05 eq) were added to the solution. Reaction mixture was stirred for 48–72 h at room temperature. Progress of the reaction was monitored by TLC using dichloromethane and methanol as eluent. The solvent was evaporated under reduced pressure. Crude residue was purified by column chromatography on silica gel eluting with dichloromethane/methanol (200:1).

##### 7,7′-(4,4′-(1,3-Phenylene)bis(1H-1,2,3-triazole-4,1-diyl))bis(4-methyl-2H-chromen-2-one) (**2h**)

Compound **2h** was synthesised following described method from azidocoumarin **1** (1.0 eq) and 1,3-diethynylbenzene (0.5 eq). After stirring for 48 h at r.t. and purification, compound **2h** as yellow powder was isolated (20.4 mg, 51%); m.p. 247–253 °C.

^1^H NMR (300 MHz, DMSO-*d*_6_): *δ*/ppm = 9.56 (s, 2H), 8.03–8.01 (m, 6H), 7.52–7.51 (m, 4H), 6.49 (s, 2H), 1.33 (s, 6H). ^13^C NMR (75 MHz, DMSO-*d*_6_): *δ*/ppm = 159.9, 154.1, 153.2, 147.1, 138.9, 132.0, 130.9, 130.1, 128.9, 127.9, 126.2, 124.6, 123.0, 120.1, 115.9, 115.2, 115.1, 18.6. HRMS (MALDI TOF/TOF) *m*/*z* calcd for C_30_H_20_N_6_O_4_ [M]^+^: 528.1546, found [M]^+^: 528.1550.

##### 7,7′-(4,4′-(1,4-Phenylene)bis(1H-1,2,3-triazole-4,1-diyl))bis(4-methyl-2H-chromen-2-one) (**2i**)

Compound **2i** was prepared from azidocoumarin **1** (1.0 eq) and 1,4-diethynylbenzene (0.5 eq). After stirring for 72 h at r.t. and column chromatography, compound **2i** (13.7 mg, 35%) was isolated as yellow powder; m.p. 255–261 °C. 

^1^H NMR (300 MHz, DMSO-*d*_6_): *δ*/ppm = 9.54 (s, 2H), 8.03 (s, 4H), 7.98–7.62 (m, 6H), 6.50 (s, 2H), 1.23 (s, 6H). ^13^C NMR (75 MHz, DMSO-*d*_6_): *δ*/ppm = 159.9, 154.1, 153.2, 138.9, 133.0, 130.8, 127.9, 126.0, 122.1, 120.9, 120.1, 115.9, 115.2, 107.9, 18.6. HRMS (MALDI TOF/TOF) *m*/*z* calcd for C_30_H_20_N_6_O_4_ [M]^+^: 528.1546, found [M]^+^: 528.1550. 

### 3.3. Spectroscopic Characterisation

Spectrophotometric absorbance and fluorescence measurements were carried out using 1.0 × 10^–5^ M solutions of compounds. Measurements were conducted by diluting stock solutions of **2** and **4** in DMSO due to the good solubility of all compounds in this solvent.

UV-Vis absorption spectra were recorded, against the solvent, at (25 ± 0.1) °C, using a Varian Cary spectrophotometer in double-beam mode. Quartz cells of 1 cm path length were used throughout. The wavelength range covered was from 200 to 500 nm. Fluorescence measurements were carried out on a Varian Cary Eclipse fluorescence spectrophotometer at 25 °C using 1 cm path quartz cells. Excitation wavelengths were determined from absorbance maxima. Emission spectra were recorded from 200 to 700 nm. Relative fluorescence quantum yields were determined according to Miller using Equation (1):

(1) $$\Phi_{x}=\Phi_{s}\times A_{s}D_{x}n_{x}^{2}/A_{x}D_{s}n_{s}^{2}$$

where *Φ* is the emission quantum yield, *A* is the absorbance at the excitation wavelength, *D* is the area under the corrected emission curve and *n* is the refractive index of the solvents used. The subscripts *s* and *x* refer to the standard and to the unknown, respectively. The standard employed was quinine sulphate with a published fluorescence quantum yield of 0.54 [39]. 

### 3.4. Antiproliferative Activity In Vitro

The experiments were carried out on several human cell lines, as follows: HCT116 (colon carcinoma), H 460 (lung carcinoma) and MCF-7 (breast carcinoma), HeLa (cervical carcinoma), human T-lymphocyte cells (CEM) and HMEC-1 (dermal microvascular endothelial cell) according to the previously published experimental procedure [40,41]. Briefly, the cells were grown in DMEM medium with the addition of 10% foetal bovine serum (FBS), 2 mM L-glutamine, 100 U/mL penicillin and 100 µg/mL streptomycin, and cultured as monolayers at 37 °C in a humidified atmosphere with 5% CO_2_. HCT116, H 460 and MCF-7 cells were seeded at 2 × 10^3^ cells/well in a standard 96-well microtiter plates and left to attach for 24 h. Next day, test compound was added in five serial 10-fold dilutions. The cell growth rate was evaluated after 72 h of incubation, using MTT assay. Obtained results were expressed as IC_50_ value which stands for the concentration of the compound necessary for 50% of growth inhibition. The IC_50_ values were calculated from concentration-response curve using linear regression analysis by fitting the test concentrations that give percentage of growth (PG) values above and below the reference value (i.e., 50%). However, if for a given cell line all of the tested concentrations produce PGs exceeding the respective reference level of effect (e.g., PG value of 50), then the highest tested concentration is assigned as the default value, which is preceded by a “>” sign. Each test was performed in quadruplicate in at least two individual experiments.

HeLa and HMEC-1 cells were seeded in 48-well plates at 10,000 cells/well. After 24 h, different concentrations of the compounds were added. After 3 days of incubation, the cells were trypsinized and counted in a Coulter counter. Suspension cells (human T-cell leukaemia Cem cells) were seeded in 96-well plates at 60,000 cells/well in the presence of different concentrations of compounds, allowed to proliferate for 96 h, and counted in a Coulter counter. The 50% inhibitory concentration (IC_50_) was defined as the compound concentration required reducing cell proliferation by 50%.

## 4. Conclusions

In this work we present the synthesis of coumarin-triazole conjugates using the copper(I)-catalysed Huisgen 1,3-dipolar cycloaddition. The synthesised compounds were characterised by ^1^H-NMR, ^13^C-NMR and HRMS. The synthesised compounds were tested against several human cancer cell lines (HCT116, MCF-7, H 460, CEM and HeLa) and non-tumour HMEC-1 cell line. The antiproliferative assay demonstrated moderate to pronounced activity of the compounds against some cancer cells, particularly against breast adenocarcinoma (MCF-7). Amongst coumarin-triazole-aryl hybrids, compound **2b** with an amino substituent at the phenyl moiety showed the most pronounced activity towards MCF-7 cancer cells in submicromolar range (IC_50_ = 0.6 µM) and enhanced activity against HCT116 cells with IC_50_ = 3 µM. 1,3-Bis(coumarin-1,2,3-trazolyl)benzene **2h** strongly inhibited growth of MCF-7 cells (IC_50_ = 0.3 µM). Its 1,4 structural congener showed somewhat reduced antiproliferative activity with IC_50_ = 1 µM against the same cancer cell line. The two coumarin-triazole-benzazole derivatives **4a** and **4b** showed very similar moderate activity against all of the cancer cell lines, whereas the presence of a benzothiazole or benzimidazole moiety had no influence on antiproliferative activity for **4a** or **4b**, respectively. Generally, the MCF-7 cell line showed the most evident sensitivity towards most of the tested compounds, while non-tumour human HMEC-1 cell line remained almost completely unaffected. Thus, further appropriate structural modification of the most effective molecular hybrids is prerequisite to deliver more potent new compounds with broad and improved biological properties.

The absorption and emission spectra of the synthesised compounds revealed some interesting optical properties. These include large Stokes shifts of the coumarin-triazole-aryl hybrids **2a**–**2g** (78–187 nm) and the dual emission peaks of the benzazole derivatives **4a** and **4b**. These spectroscopic properties suggest that the molecules may also be suitable for use as molecular probes or in bioimaging. Further detailed photophysical studies of the synthesised compounds are underway.

## Figures and Tables

**Table 1:** IC_50_ values (μM) of tested compounds against HCT116, MCF-7, H 460, CEM, HeLa HMEC-1 cells.

Compound	IC_50_^a^ (μM)							
	HCT116	MCF-7	H 460	CEM		HeLa		HMEC-1
**2a**	≥100	23 ± 9	≥100	>100		>100		>100
**2b**	3 ± 2	0.6 ± 0.1	≥100	>100		>100		>100
**2c**	≥100	21 ± 0.03	≥100	58 ± 7		88 ± 8		>100
**2d**	36 ± 3	≥100	≥100	nd ^b^				
**2e**	27 ± 0.06	32 ± 4	85 ± 13.5					
**2f**	22 ± 21	≥100	≥100					
**2g**	77 ± 7	≥100	≥100					
**2h**	9 ± 3	0.3 ± 0.2	29 ± 3	>100	>100		>100	
**2i**	7 ± 3	1 ± 0.1	≥100	>100	≥100		>100	
**4a**	31 ± 6	3 ± 2	21 ± 7	≥100	79 ± 36		66 ± 4	
**4b**	31 ± 5	19 ± 3	23 ± 10	74 ± 37	25 ± 0		69 ± 8	
**5-FU ^c^**	4 ± 1	14 ± 0.3	3 ± 0.3	nd				
**etoposide**	5 ± 2	1 ± 0.7	0.1 ± 0.04					

^a^ IC_50_; the concentration that causes 50% growth inhibition, ^b^ nd; not determined, ^c^ 5-FU; 5-fluorouracil.

**Table 2:** Absorption and emission data of studied compounds in DMSO.

Compound	2a	2b	2c	2d	2e	2f	2g	2h	2i	4a	4b
*λ*_abs/_nm	328	284 325(sh)	296 325(sh)	328	327	331	300 325	292 326	278 326	285 292 325(sh)	287 294 325(sh)
*ε* × 10^3^/dm^3^ mol^−1^ cm^−1^	28.7	39.7 15.3	41.0	15.1	23.2	18.1	11.4 13.5	12.2 14.4	53.7 39.3	37.6 36.3 22.5	29.3 29.2 17.6
*λ*_emis_/nm	406	412	418	455	401	517	411	394	415	416 350	418 347
Rel. Fluo. Int.	292.7	92.6	4.3	593.3	229.3	43.1	63.0	451.4	119.3	362.6 218.9	396.8 235.1
Stokes shift/nm	78	129 87	123	126	73	187	84	64	88	91 124	91 122
*Φ*	0.212	0.035	0.004	0.445	0.136	0.058	0.021	0.127	0.029	0.018	0.025

Absorption and fluorescence spectra were recorded at compound concentration *c* = 1 × 10^−5^ M, *λ*_exc_ = *λ*_abs,max_, slit width = 5–5. Stock solutions of compounds were prepared in DMSO.

## Data Availability

The data presented in this study are available in this article and the Supplementary Materials.

## Supplementary Materials

The following supporting information can be downloaded online. Table S1: ^1^H and ^13^C NMR and HRMS spectral data and melting points for 1,2,3-triazolyl-coumarin derivatives **2a**–**i** and **4a**–**b**, Figures S1–S14: ^1^H and ^13^C NMR spectra of **1**, **2a**–**i**, **3a**–**b**, **4a**–**b**, Figures S15–S25: HRMS spectra of compounds **2a**–**i** and **4a**–**b**.

## Sample Availability

Samples of the compounds are available from the authors.
