# Supplementary material for: Design, Synthesis, Spectroscopic Characterisation and In Vitro Cytostatic Evaluation of Novel Bis(coumarin-1,2,3-triazolyl)benzenes and Hybrid Coumarin-1,2,3-triazolyl-aryl Derivatives
Source: Molecules. 2022 Jan 19;27(3):637. doi: 10.3390/molecules27030637 (PMC8840664; doi:10.3390/molecules27030637)

Supplementary Materials

Design, Synthesis, Spectroscopic Characterisation and *In Vitro* Cytostatic Evaluation of Novel Bis(Coumarin-1,2,3-Triazolyl)benzenes and Hybrid Coumarin-1,2,3-Triazolyl-Aryl Derivatives

Kristina Pršir [1], Ema Horak [2], Marijeta Kralj [3], Lidija Uzelac [3], Sandra Liekens [4], Ivana Murković Steinberg [1] and Svjetlana Krištafor [1,*]

[1] *Department of General and Inorganic Chemistry, Faculty of Chemical Engineering and Technology, University of Zagreb, Marulićev trg 19, 10000 Zagreb, Croatia; kbobanov@fkit.hr (K.P.); imurkov@fkit.hr (I.M.S.)*

[2] *Fidelta Ltd., Prilaz baruna Filipovića 29, 10000 Zagreb, Croatia; Ema.Horak@fidelta.eu (E.H.)*

[3] *Division of Molecular Medicine, Ruđer Bošković Institute, Bijenička cesta 54, 10000 Zagreb, Croatia; Marijeta.Kralj@irb.hr (M.K.); Lidija.Uzelac@irb.hr (L.U.)*

[4] *KU Leuven, Department of Microbiology and Immunology, Rega Institute for Medical Research, Laboratory of Virology and Chemotherapy, 3000 Leuven, Belgium; Sandra.liekens@kuleuven.be (S.L.)*

*Correspondence: prekupec@fkit.hr (S.K.)

Table S1. Structures, yields, ^{1}H and ^{13}C NMR and HRMS spectral data and melting points for 1,2,3-triazolyl-coumarin derivatives **2a–i** and **4a–b**

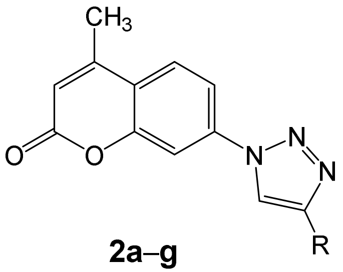

2a–g
4a–b

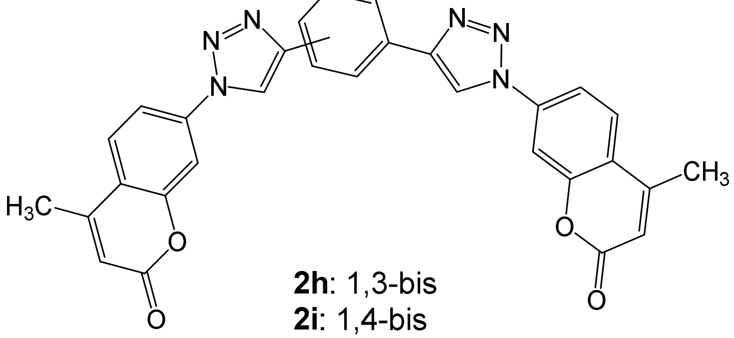

2h: 1,3-bis
2i: 1,4-bis

**Compd**	**2a**	**2b**	**2c**	**2d**	**2e**	**2f**	**2g**	**2h**	**2i**	**4a**	**4b**
**R**	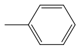	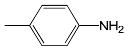	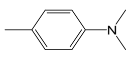	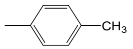	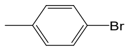	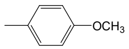	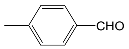	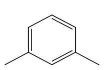	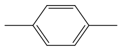	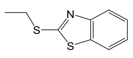	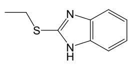
η / %	25	17	15	41	59	51	11	51	35	55	68
**^{1}H NMR δ/ppm**	9.49 (s, 1H),<br>8.05 (s, 3H),<br>7.97 (d, 2H, *J* = 7.1 Hz),<br>7.56-7.51 (m, 2H),<br>7.45-7.40 (m, 1H),<br>6.52 (s, 1H),<br>2.74 (s, 3H)	9.17 (s, 1H),<br>8.01 (s, 3H),<br>7.60 (d, 2H, *J* = 8.4 Hz),<br>6.67 (d, 2H, *J* = 8.5 Hz),<br>6.49 (s, 1H),<br>5.35 (s, 2H)<br>2.27 (s, 3H)	9.25 (s, 1H),<br>8.02 (s, 3H),<br>7.76 (d, 2H, *J* = 8.5 Hz),<br>6.84 (d, 2H, *J* = 8.7 Hz),<br>6.49 (s, 1H),<br>2.97 (s, 6H),<br>1.23 (s, 3H)	9.42 (s, 1H),<br>8.03 (s, 3H),<br>7.84 (d, 2H, *J* = 8.1 Hz),<br>7.33 (d, 2H, *J* = 7.9 Hz),<br>6.50 (s, 1H),<br>2.36 (s, 3H),<br>1.23 (s, 3H)	9.53 (s, 1H),<br>8.03 (s, 3H),<br>7.91 (d, 2H, *J* = 8.5 Hz),<br>7.74 (d, 2H, *J* = 8.5 Hz),<br>6.51 (s, 1H),<br>2.73 (s, 3H)	9.63 (s, 1H),<br>8.03 (s, 3H),<br>7.88 (d, 2H, *J* = 8.8 Hz),<br>7.09 (d, 2H, *J* = 8.8 Hz),<br>6.50 (s, 1H),<br>3.82 (s, 3H)<br>2.73 (s, 3H)	10.12 (s, 1H),<br>9.66 (s, 1H),<br>8.48 (s, 1H),<br>8.28 (d, 1H, *J* = 7.7 Hz),<br>8.05 (s, 3H),<br>7.97 (d, 1H, *J* = 7.6 Hz),<br>7.81-7.76 (m, 1H),<br>6.51 (s, 1H),<br>2.73 (s, 3H)	9.56 (s, 2H),<br>8.03-8.01 (m, 6H),<br>7.52-7.51 (m, 4H),<br>6.49 (s, 2H),<br>1.33 (s, 6H)	9.54 (s, 2H),<br>8.03 (s, 4H),<br>7.98-7.62 (m, 6H),<br>6.50 (s, 2H),<br>1.23 (s, 6H)	9.00 (s, 1H),<br>8.03-7.92 (m, 5H),<br>7.49 (t, 1H, *J* = 7.6 Hz),<br>7.40-7.35 (m, 1H),<br>6.46 (s, 1H),<br>4.82 (s, 2H)<br>2.47 (s, 3H)	12.91 (s, 1H),<br>8.95 (s, 1H),<br>7.95-7.89 (m, 3H),<br>7.55 (bs, 1H),<br>7.44 (bs, 1H),<br>7.15-7.13 (m, 2H),<br>6.47 (s, 1H),<br>4.78 (s, 2H)<br>2.46 (s, 3H)

**^{13}C NMR δ/ppm**	159.9, 154.2, 153.2, 148.1, 139.0, 130.4, 129.6, 129.0, 127.8, 125.9, 120.3, 120.0, 115.9, 115.2, 107.8, 18.6	152.3, 149.7, 149.2, 147.5, 139.2, 135.4, 127.7, 127.0, 119.7, 117.8, 117.7, 115.7, 115.0, 114.4, 107.5, 18.6	167.3, 159.9, 153.3, 150.9, 148.8, 128.4, 127.7, 126.8, 125.9, 118.1, 118.0, 115.7, 115.0, 112.8, 107.6, 29.5, 18.6	159.4, 153.7, 152.7, 147.7, 138.5, 137.9, 129.6, 127.3, 127.1, 125.3, 119.4, 115.3, 114.6, 112.7, 107.3, 20.8, 18.6	159.9, 154.1, 153.2, 147.1, 138.9, 132.6, 129.7, 127.8, 122.0, 120.7, 120.1, 115.9, 115.2, 107.9, 18.6	159.9, 159.9, 154.2, 153.2, 148.1, 139.0, 127.8, 127.8, 122.9, 119.9, 119.2, 115.8, 115.1, 115.0, 107.7, 55.7, 18.6	193.0, 168.5, 159.4, 154.6, 153.7, 152.7, 143.2, 138.5, 131.1, 131.0, 130.1, 129.8, 127.4, 125.6, 120.6, 115.4, 114.8, 107.4, 18.1	159.9, 154.1, 153.2, 147.1, 138.9, 132.0, 130.9, 130.1, 128.9, 127.9, 126.2, 124.6, 123.0, 120.1, 115.9, 115.2, 115.1, 18.6	159.9, 154.1, 153.2, 138.9, 133.0, 130.8, 127.9, 126.0, 122.1, 120.9, 120.1, 115.9, 115.2, 107.9, 18.6	156.5, 159.4, 153.6, 152.7, 152.5, 144.2, 138.3, 134.7, 127.2, 126.4, 124.6, 122.3, 121.8, 121.3, 119.5, 115.5, 114.7, 107.4, 27.2, 18.1	159.4, 153.4, 152.7, 152.6, 147.6, 138.5, 137.8, 136.5, 127.3, 127.2, 120.1, 119.4, 116.1, 115.5, 115.1, 114.7, 108.3, 107.5, 25.6, 18.0
**HRMS** *m*/*z*	calcd for $C_{18}H_{14}N_3O_2$ [M+H]$^+$: 304.1081, found [M+H]$^+$: 304.1096	calcd for $C_{18}H_{15}N_4O_2$ [M+H]$^+$: 319.1190, found [M+H]$^+$: 319.1203.	calcd for $C_{20}H_{19}N_4O_2$ [M+H]$^+$: 347.1503, found [M+H]$^+$: 347.1496	calcd for $C_{19}H_{16}N_3O_2$ [M+H]$^+$: 318.1237, found [M+H]$^+$: 318.1239	calcd for $C_{18}H_{13}BrN_3O_2$ [M+H]$^+$: 382.0186, found [M+H]$^+$: 382.0194	calcd for $C_{19}H_{16}N_3O_3$ [M+H]$^+$: 334.1186, found [M+H]$^+$: 334.1200	calcd for $C_{19}H_{14}N_3O_3$ [M+H]$^+$: 332.1030, found [M+H]$^+$: 332.1024	calcd for $C_{30}H_{20}N_6O_4$ [M]$^+$: 528.1546, found [M]$^+$: 528.1550	calcd for $C_{30}H_{20}N_6O_4$ [M]$^+$: 528.1546, found [M]$^+$: 528.1550	calcd for $C_{20}H_{14}N_4O_2S_2Na$ [M+Na]$^+$: 429.0450, found [M+Na]$^+$: 429.0471	calcd for $C_{20}H_{15}N_5O_2SNa$ [M+Na]$^+$: 412.0839, found [M+Na]$^+$: 412.0837
**m.p. / °C**	267–270	269–273	275–279	269–274	280–285	266–270	282–287	247–253	255–261	186–192	237–244

^{1}H and ^{13}C NMR spectra

Figure S1. 7-Azido-4-methyl-2H-chromen-2-one (**1**)

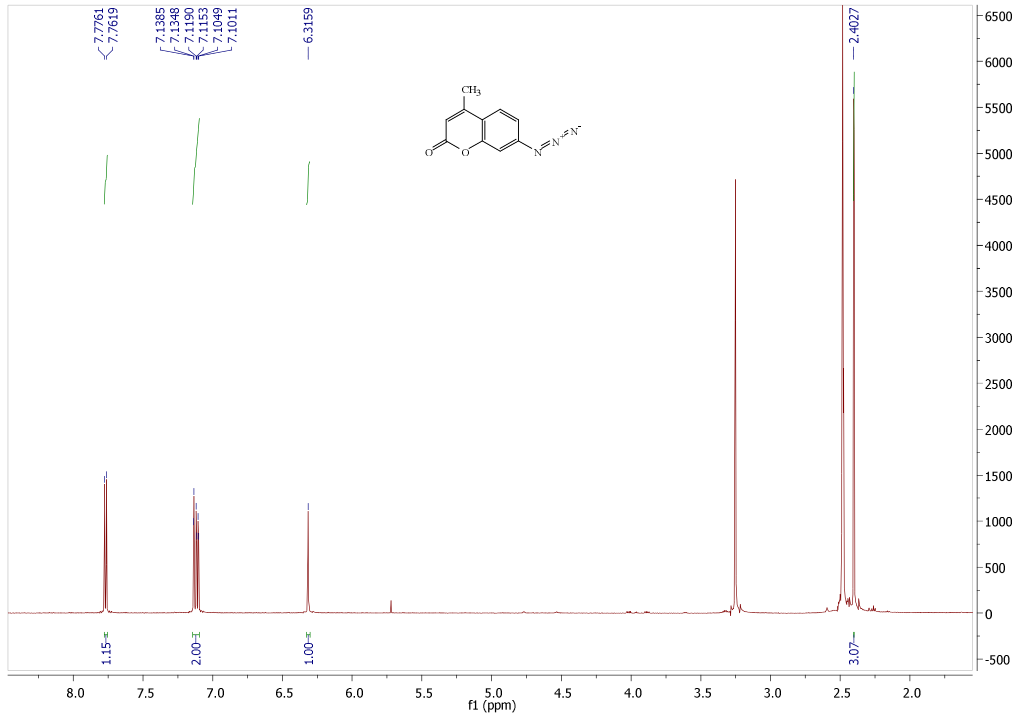

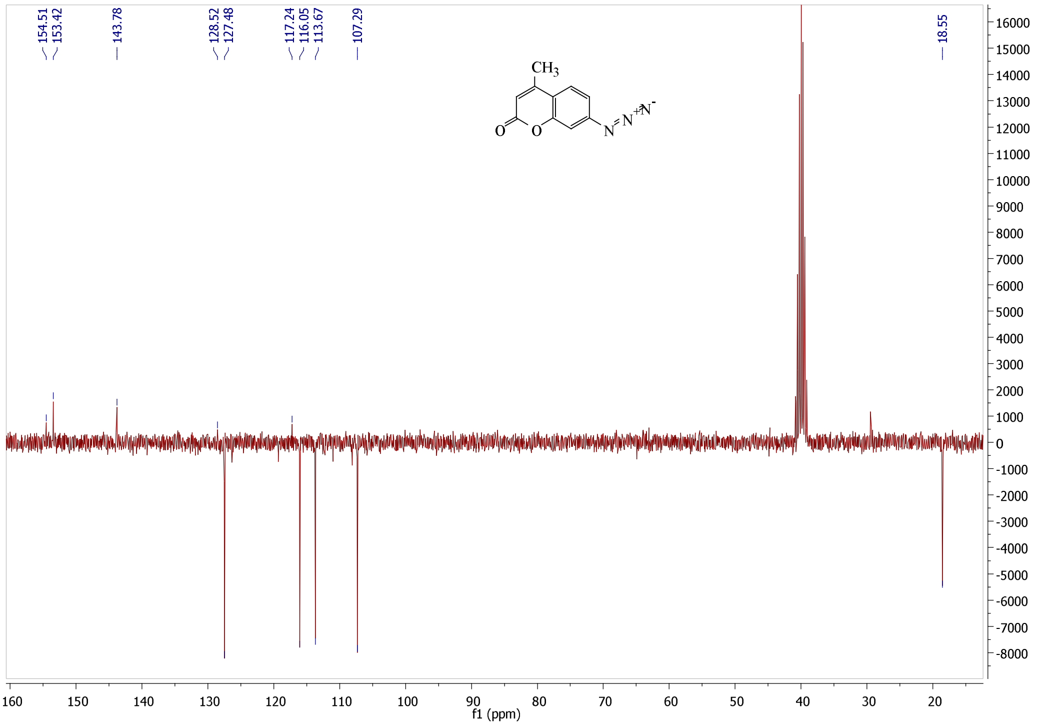

Figure S2. 4-Methyl-7-(4-phenyl-1H-1,2,3-triazol-1-yl)-2H-chromen-2-one (**2a**)

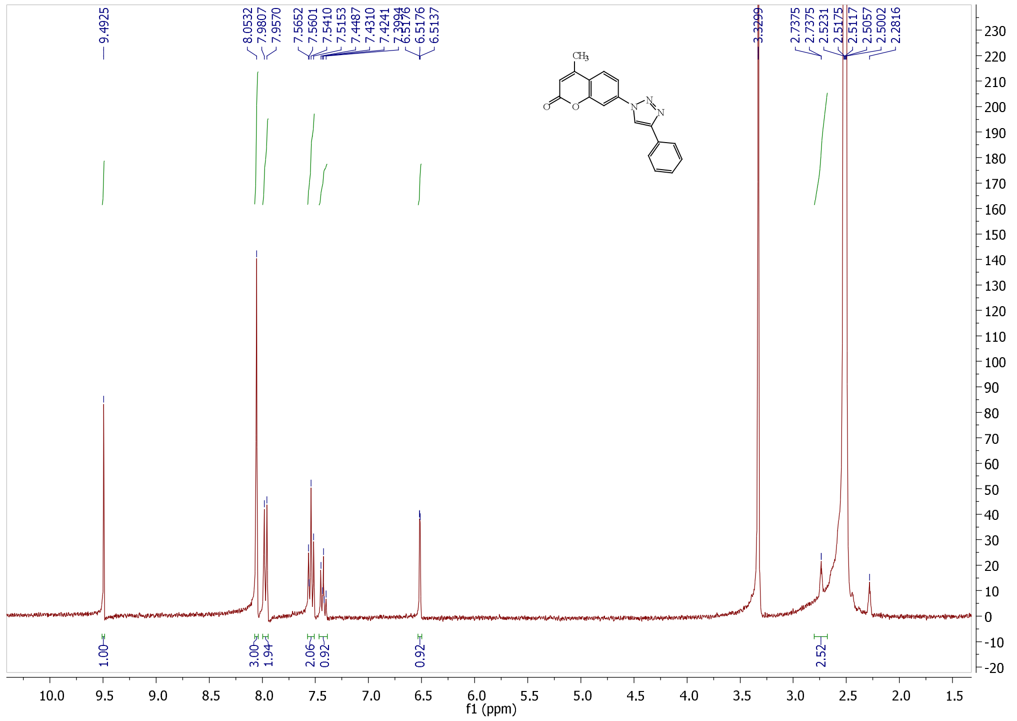

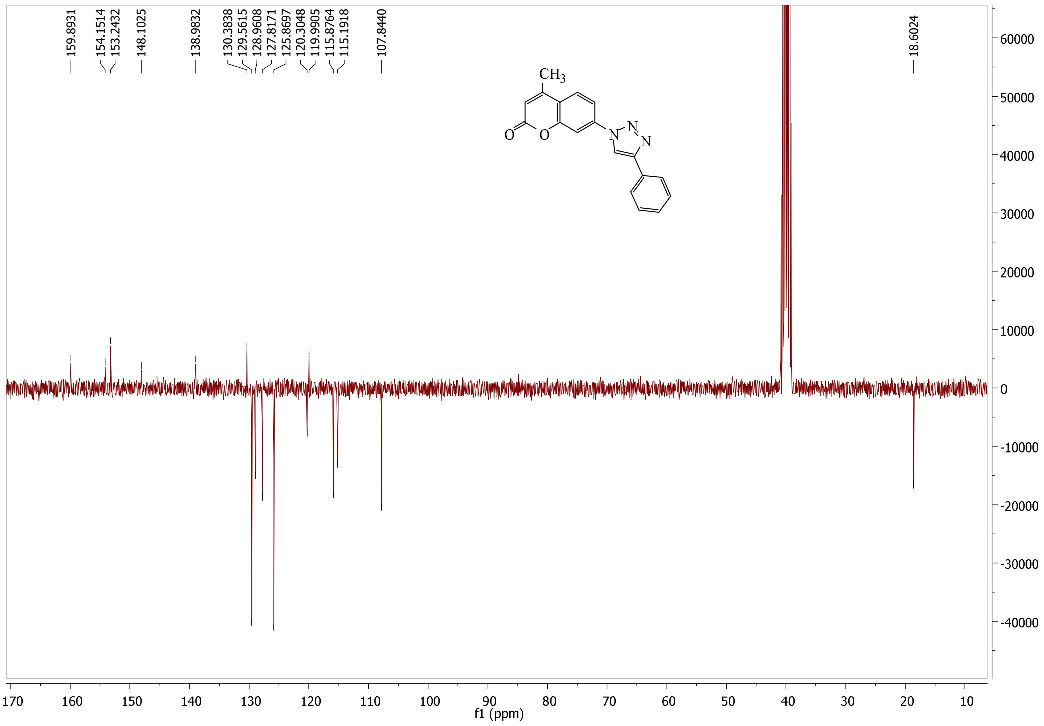

Figure S3. 7-[4-(4-Aminophenyl)-1H-1,2,3-triazol-1-yl]-4-methyl-2H-chromene-2-one (**2b**)

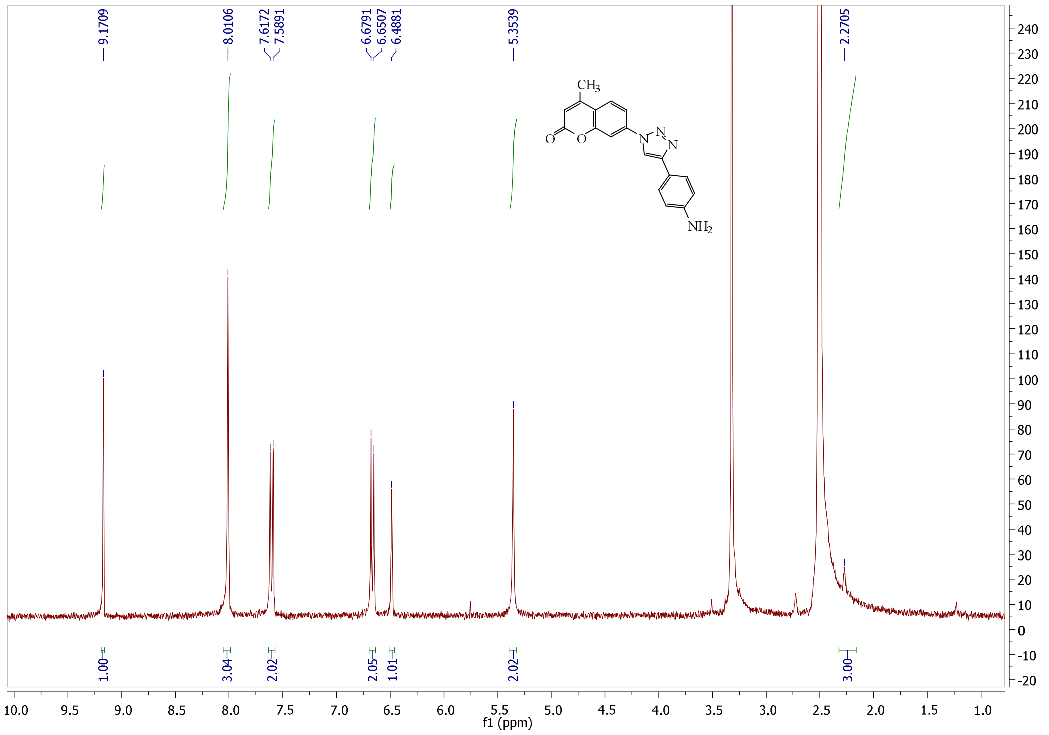

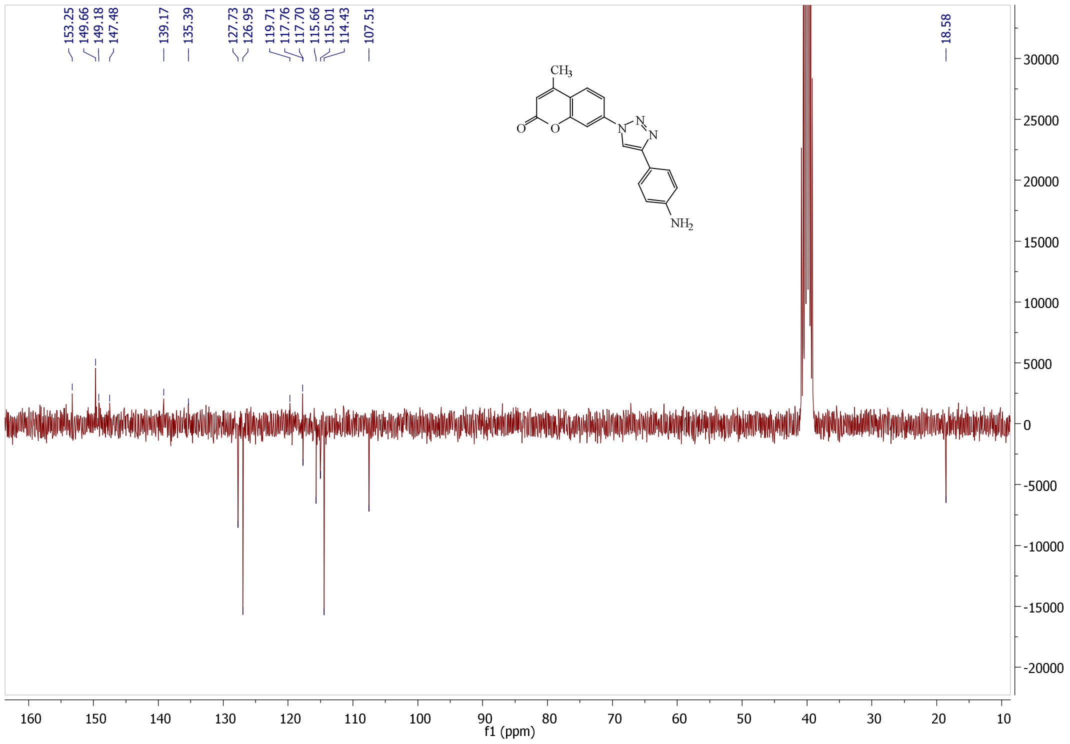

Figure S4. 7-[4-(4-(Dimethylamino)phenyl]-1H-1,2,3-triazol-1-yl)-4-methyl-2H-chromen-2-one (**2c**)

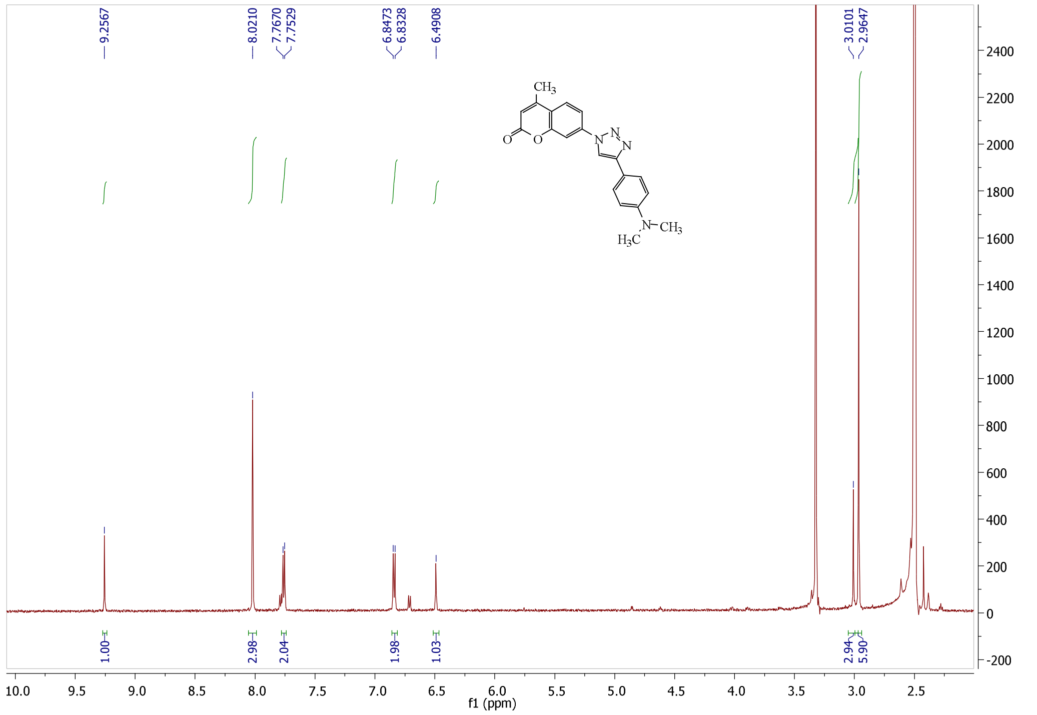

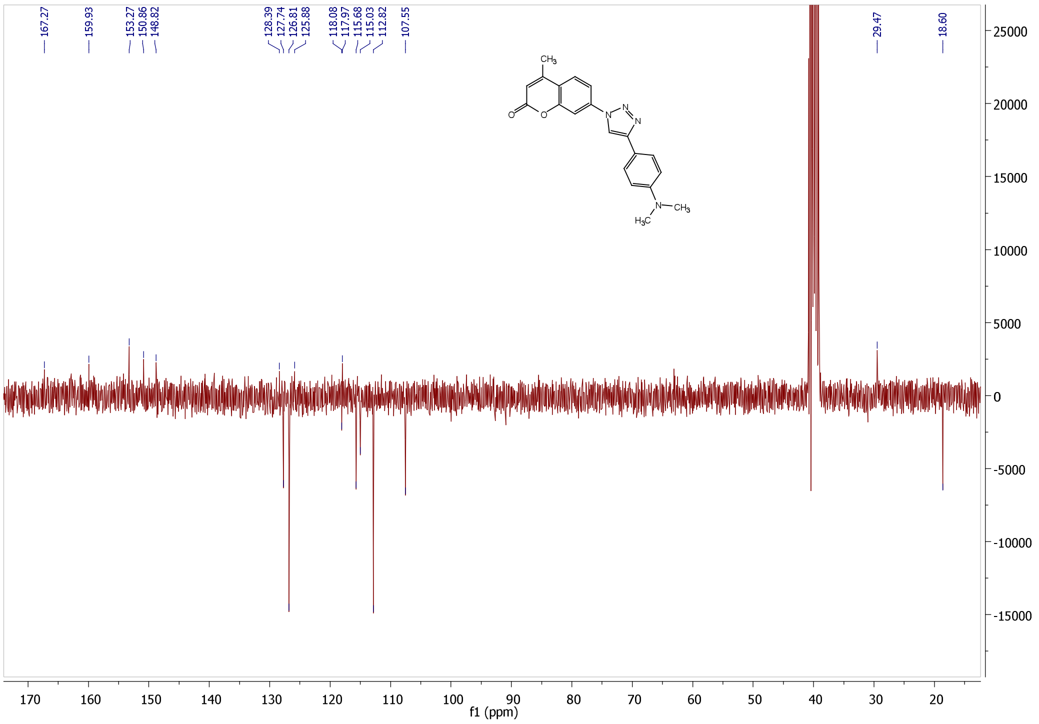

Figure S5. 4-Methyl-7-[4-(*p*-tolyl)-1H-1,2,3-triazol-1-yl]-2H-chromen-2-one (**2d**)

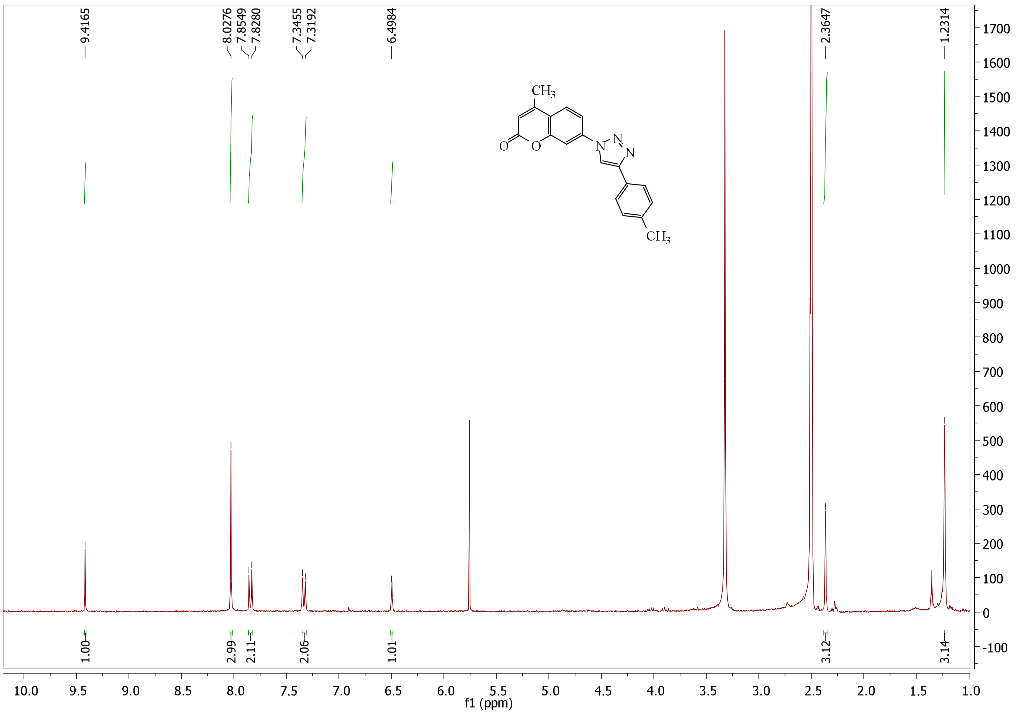

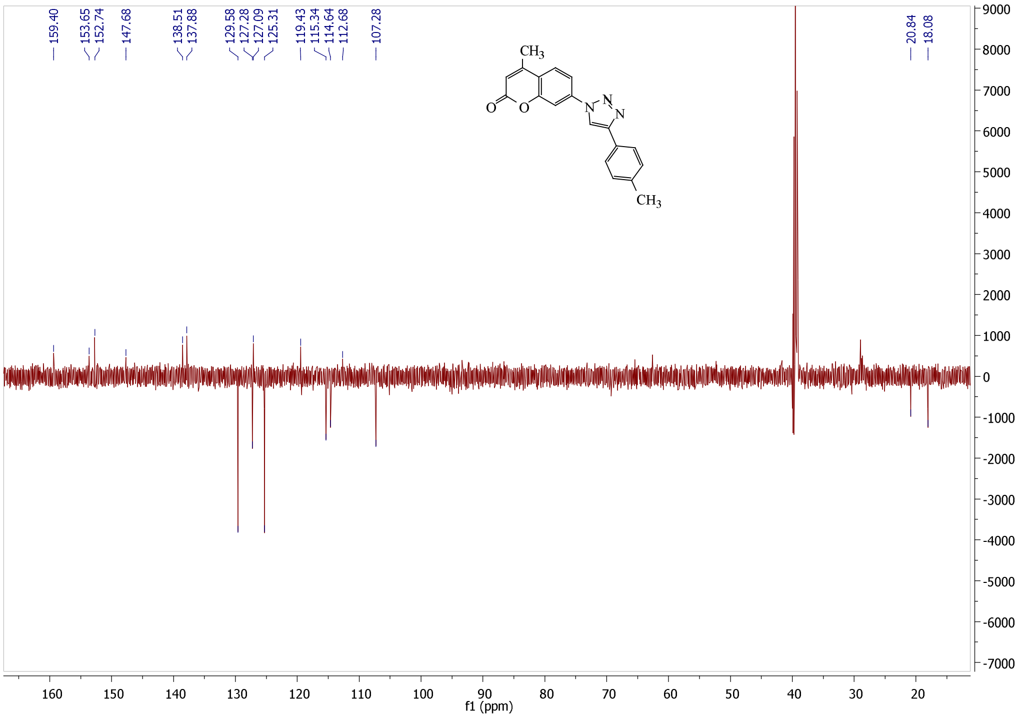

Figure S6. 7-[4-(4-Bromophenyl)-1H-1,2,3-triazol-1-yl]-4-methyl-2H-chromen-2-one (**2e**)

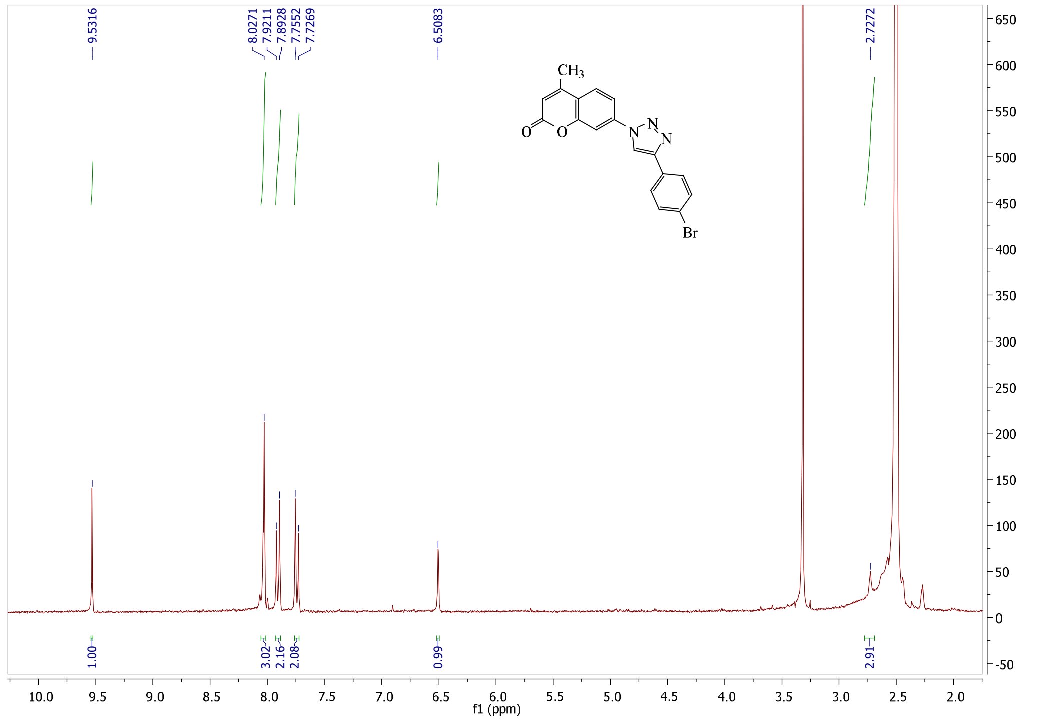

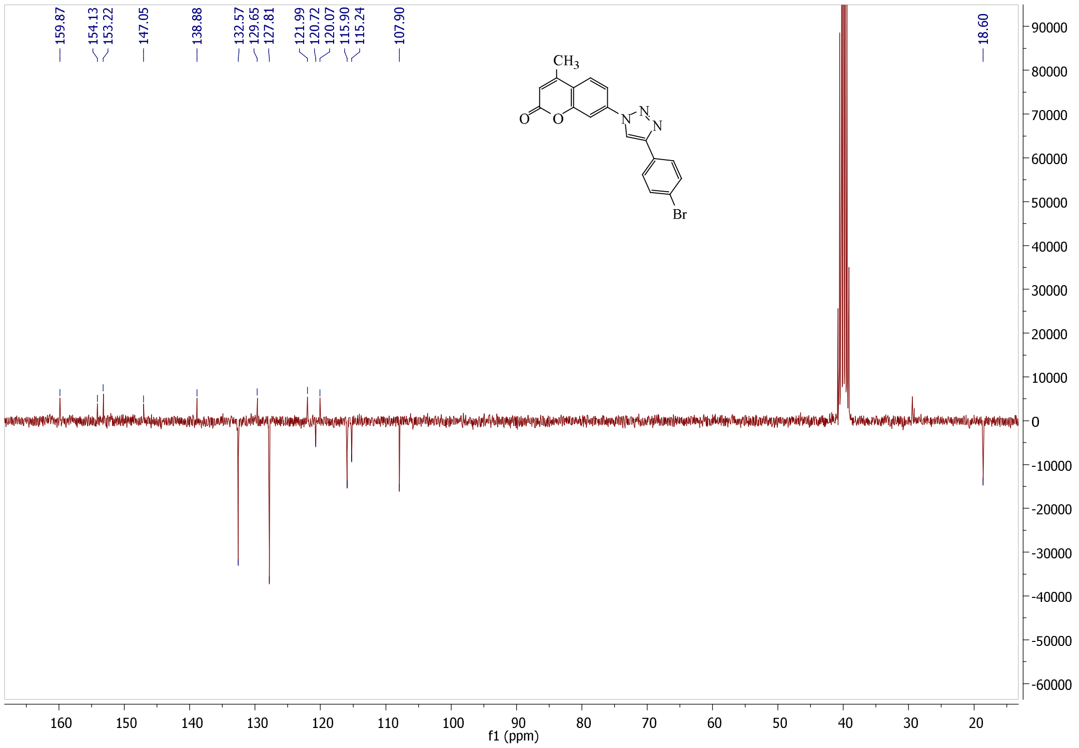

Figure S7. 7-[4-(4-Methoxyphenyl)-1H-1,2,3-triazol-1-yl]-4-methyl-2H-chromen-2-one (**2f**)

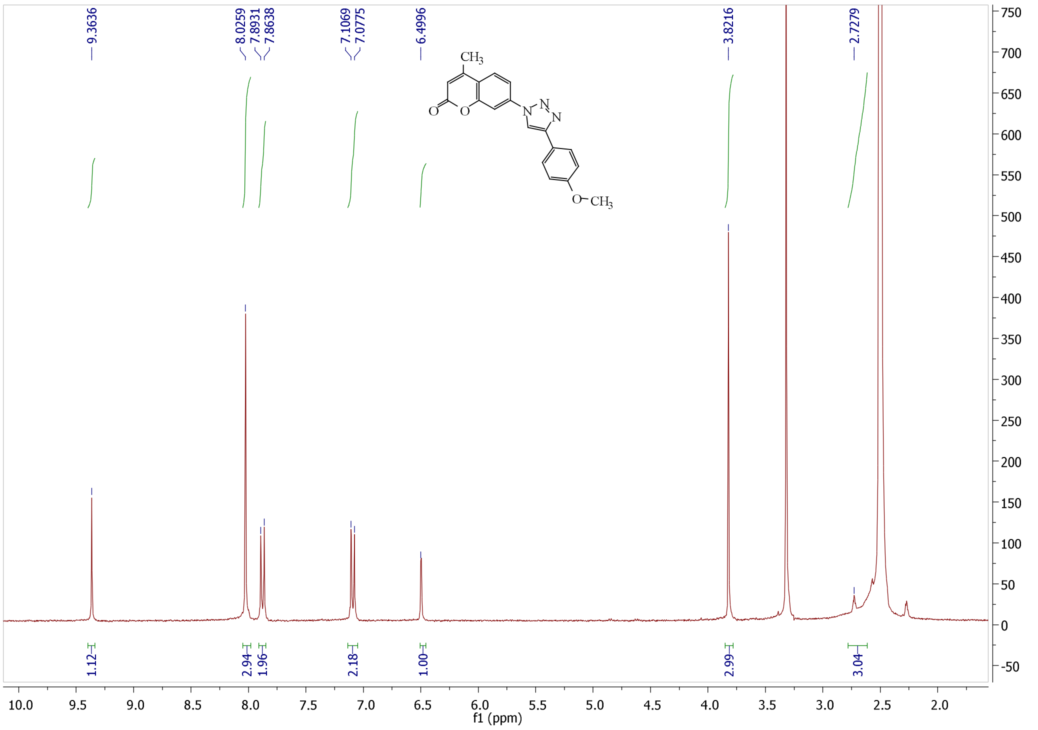

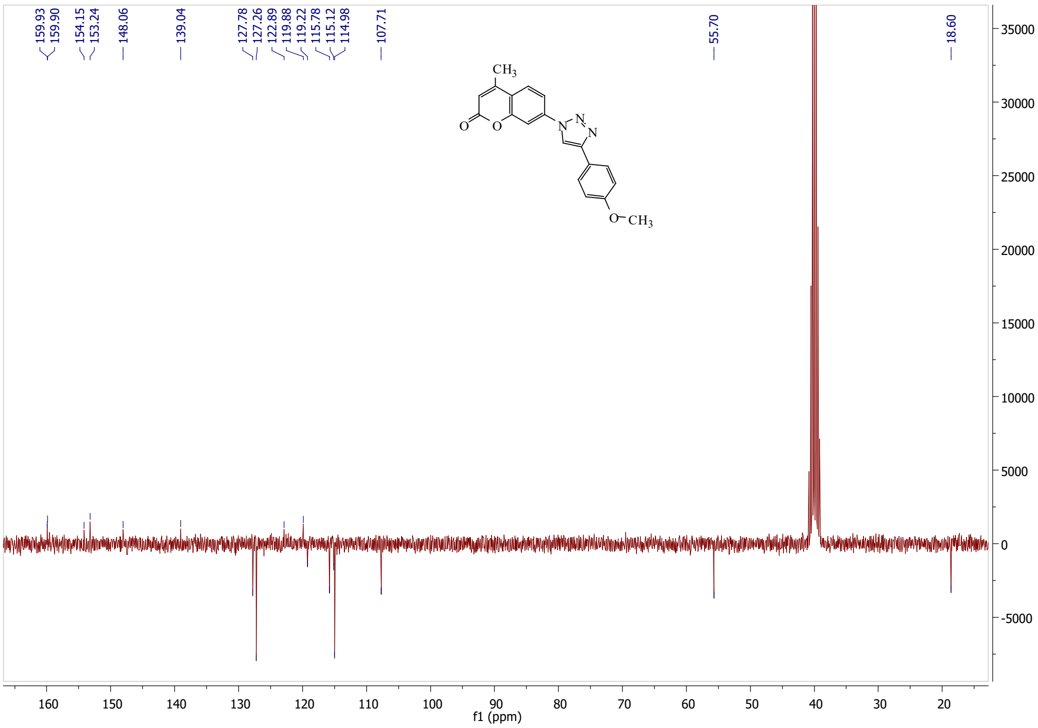

Figure S8. 4-[1-(4-Methyl-2-oxo-2H-chromen-7-yl]-1H-1,2,3-triazol-4-yl)benzaldehyde (**2g**)

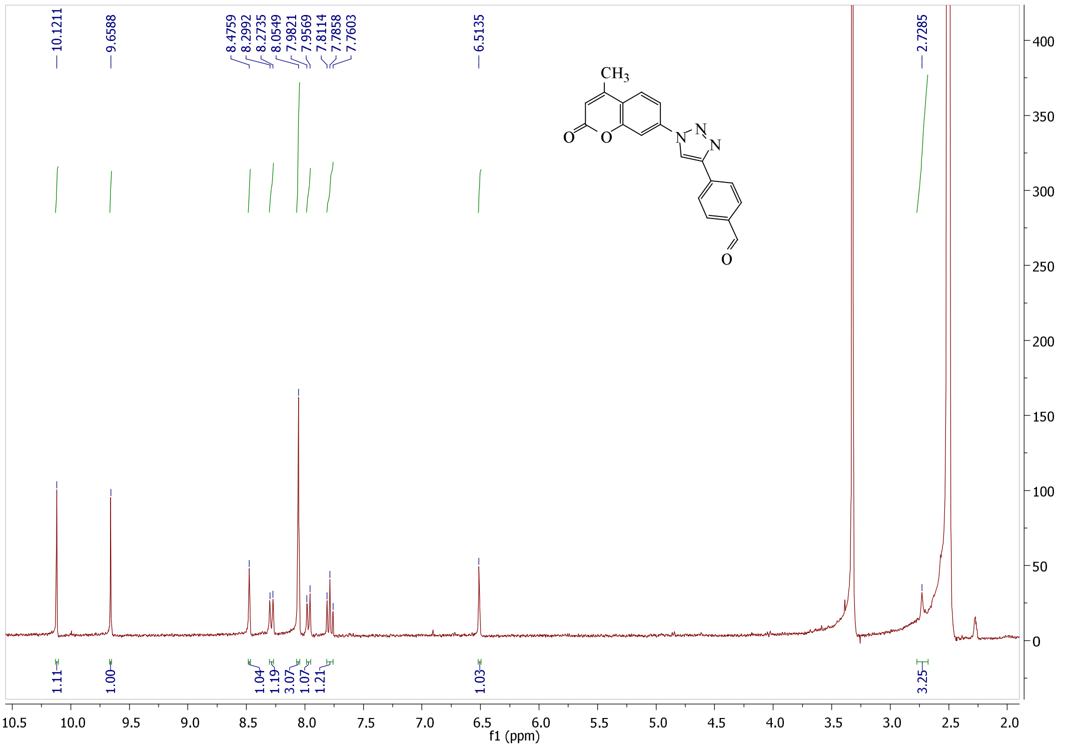

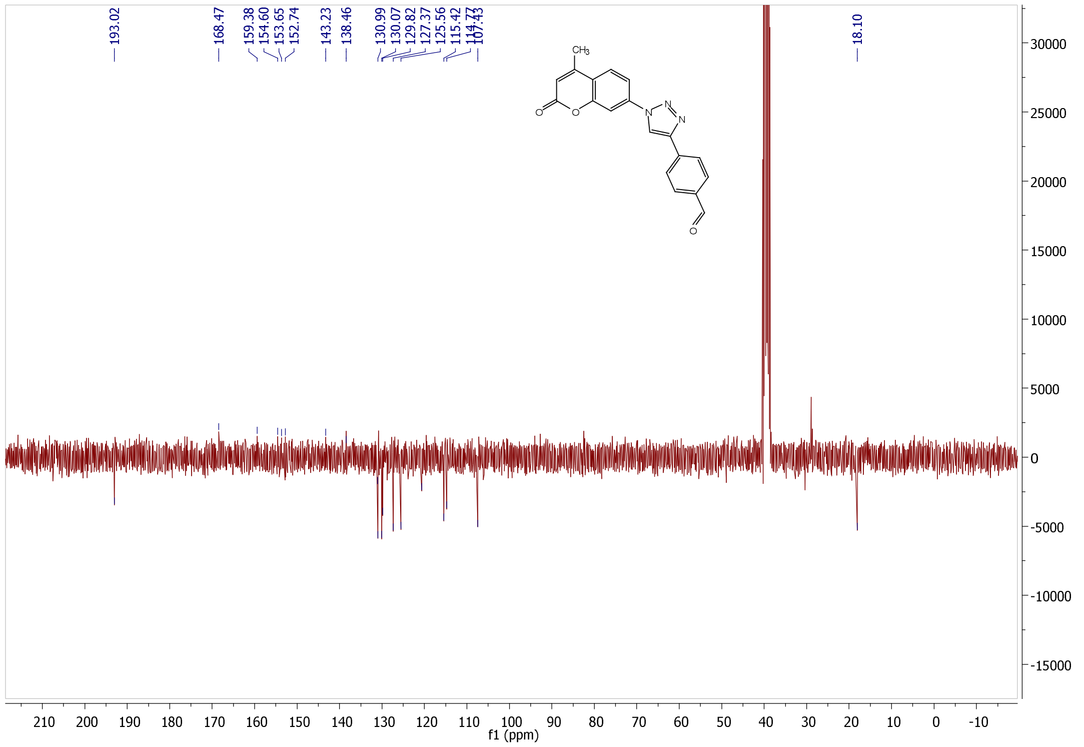

Figure S9. 7,7'-(4,4'-(1,3-phenylene)bis(1H-1,2,3-triazole-4,1-diyl))bis(4-methyl-2H-chromen-2-one) (**2h**)

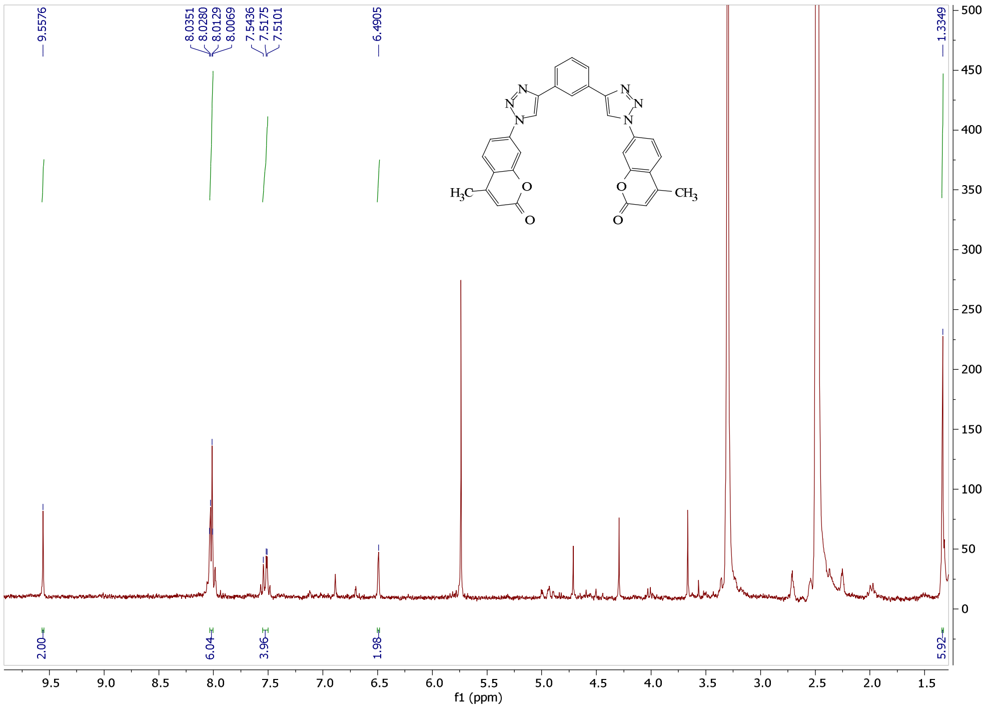

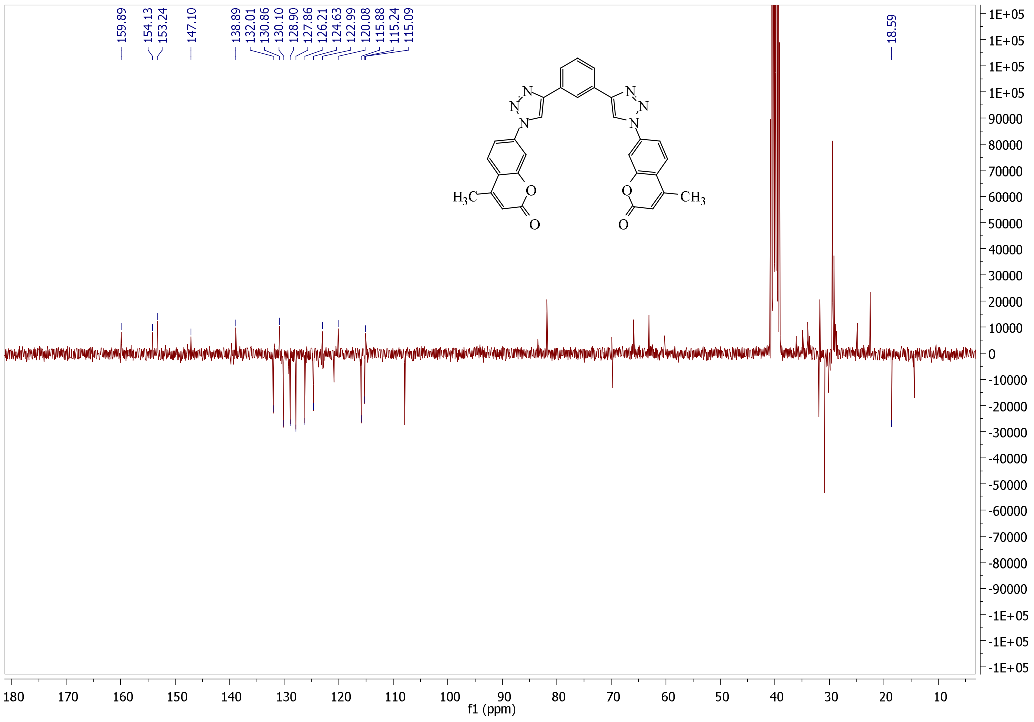

Figure S10. 7,7'-(4,4'-(1,4-phenylene)bis(1H-1,2,3-triazole-4,1-diyl))bis(4-methyl-2H-chromen-2-one) (**2i**)

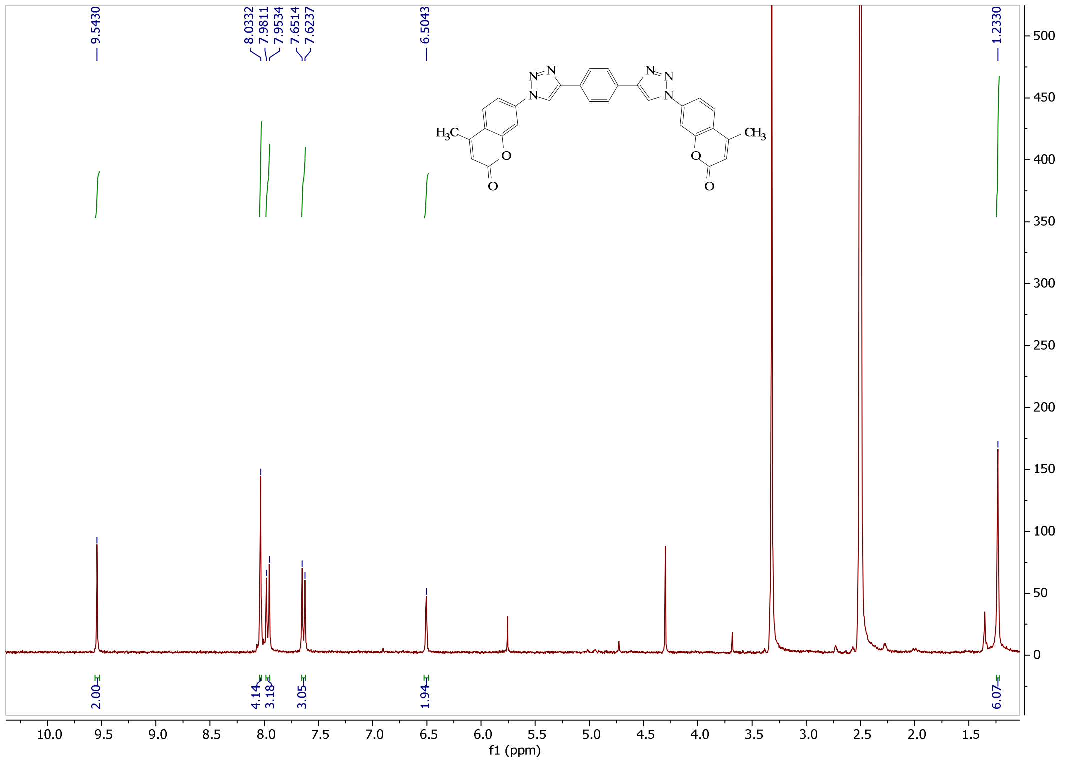

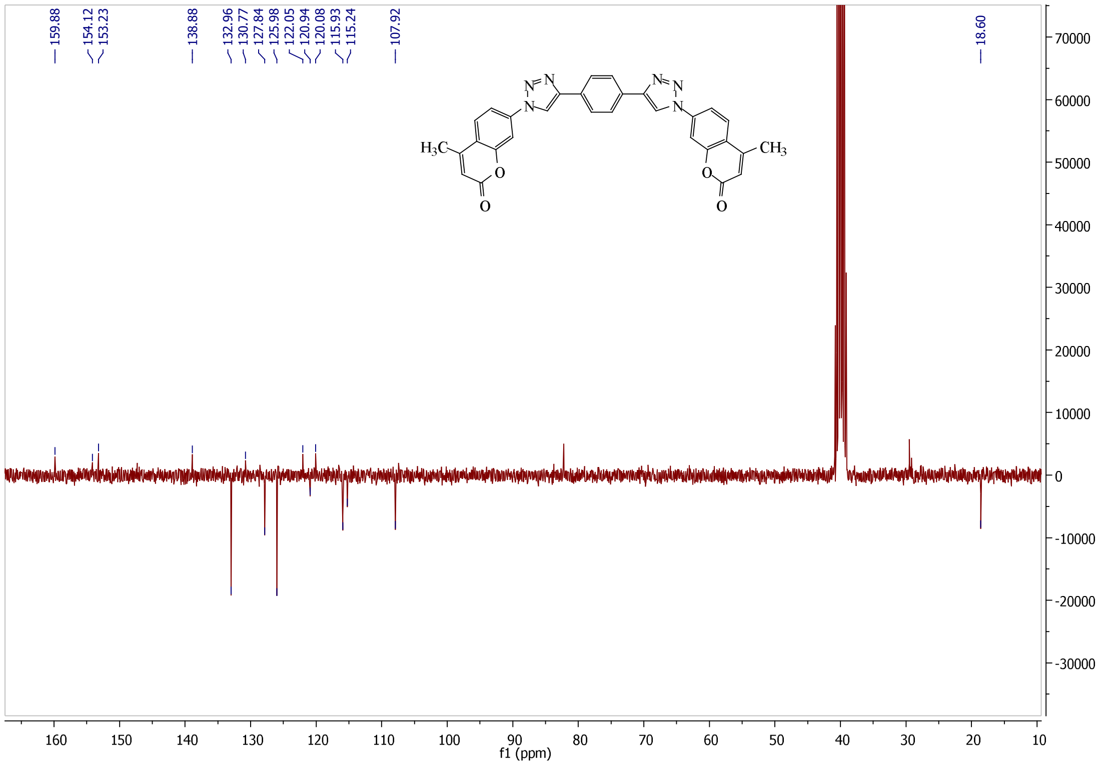

Figure S11. 2-(Prop-2-ynylthio)benzo[*d*]thiazole (**3a**)

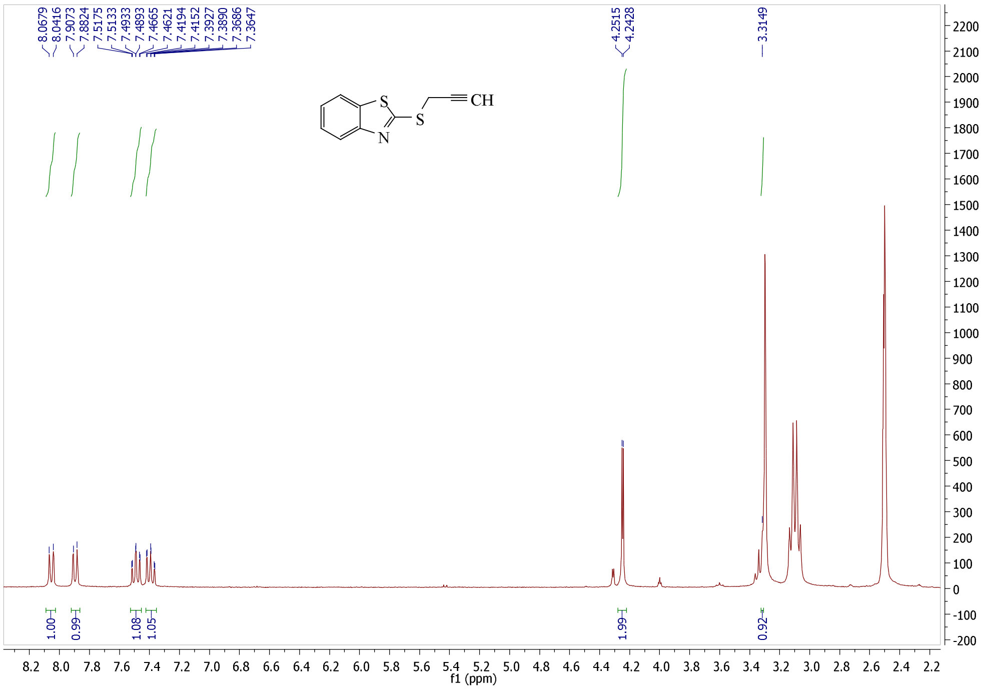

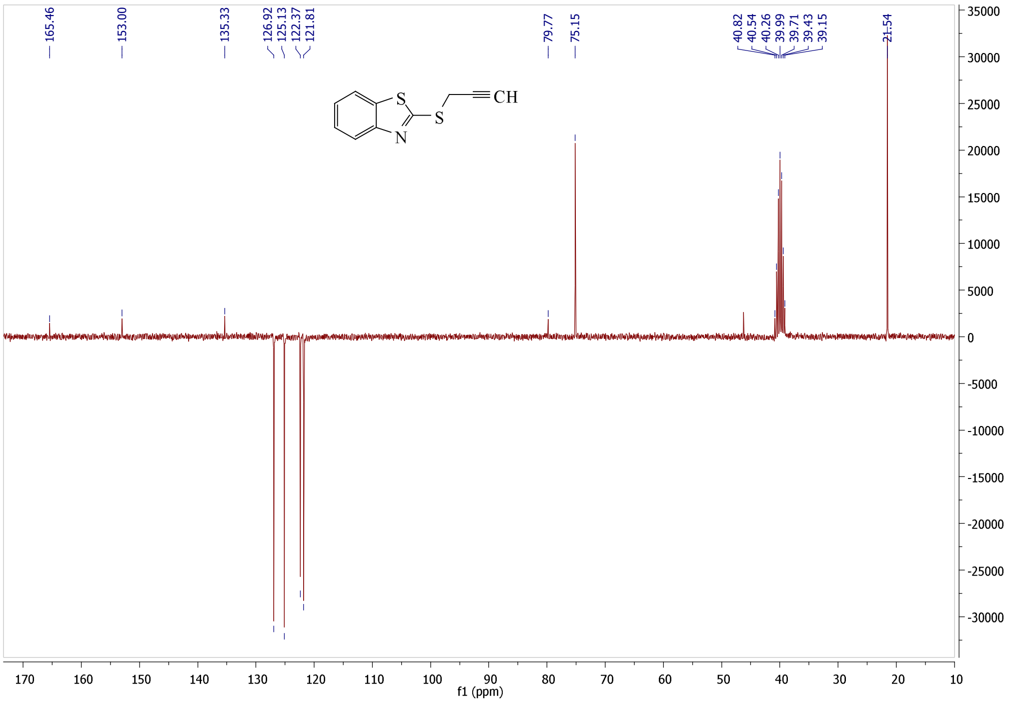

Figure S12. 2-(Prop-2-ynylthio)-1H-benzo[*d*]imidazole (**3b**)

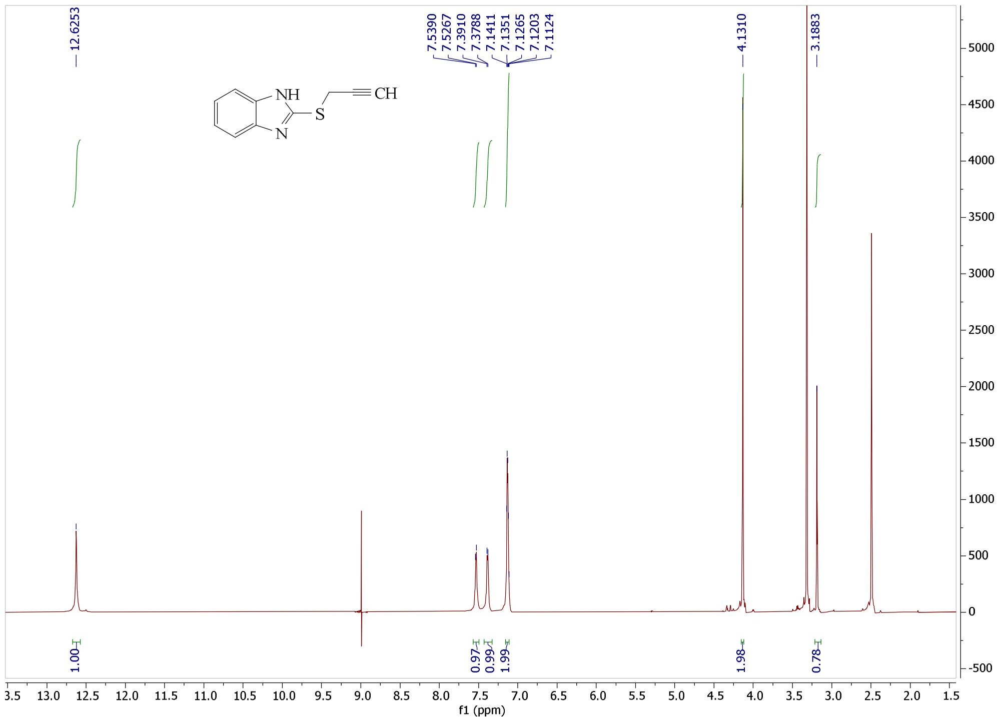

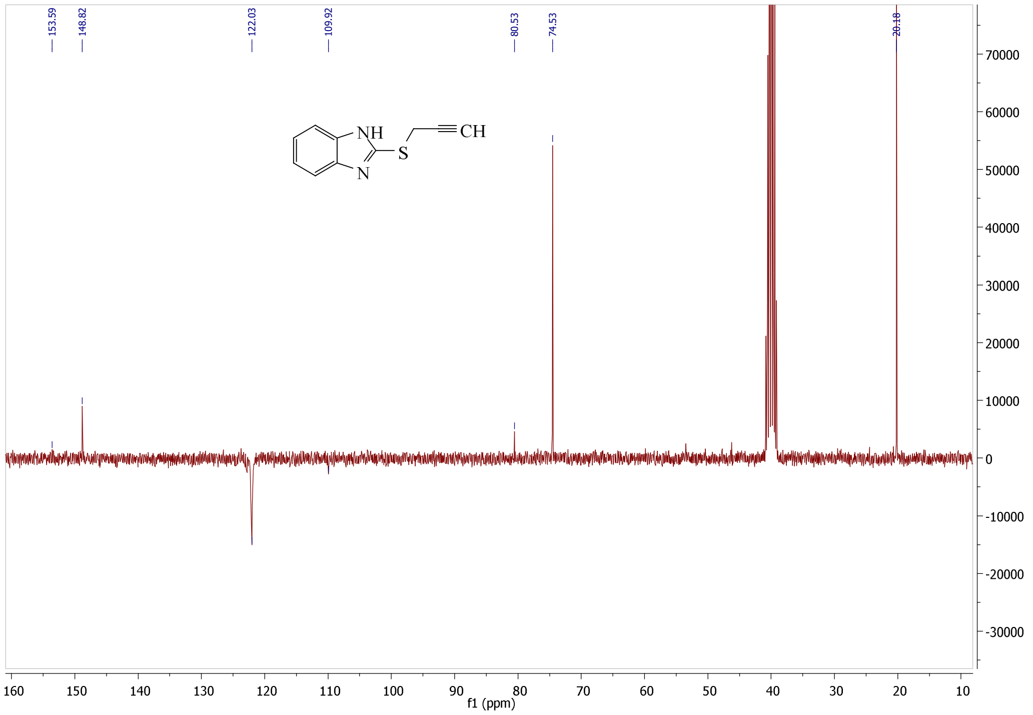

Figure S13. 7-{{4-[(Benzo[*d*]thiazol-2-yl)thio]methyl}-1H-1,2,3-triazol-1-yl}-4-methyl-2H-chromen-2-one (**4a**)

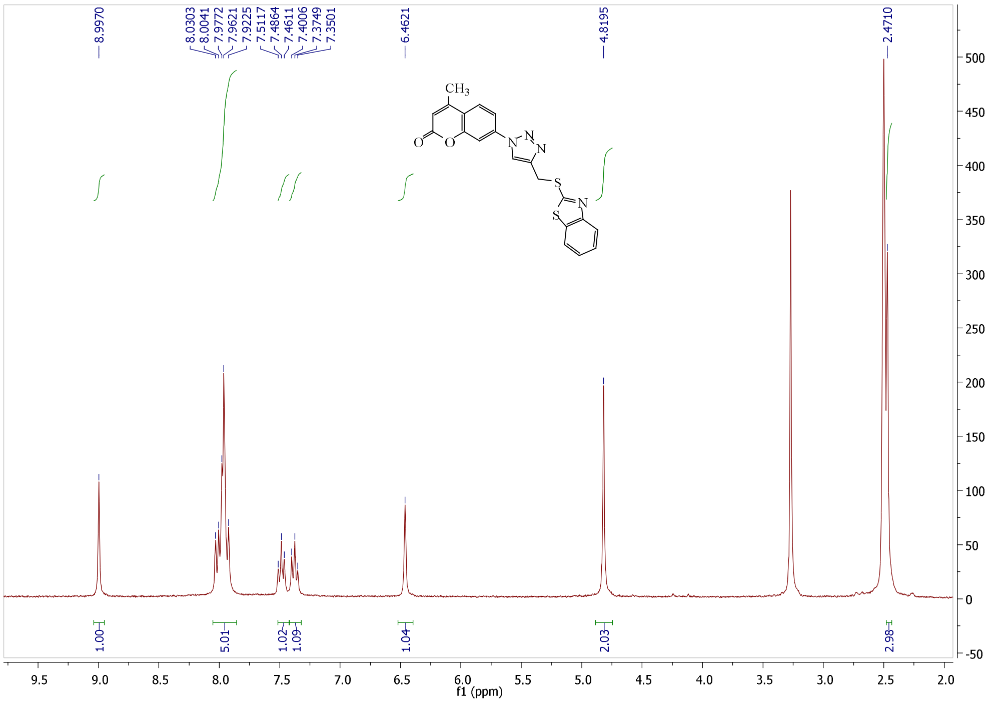

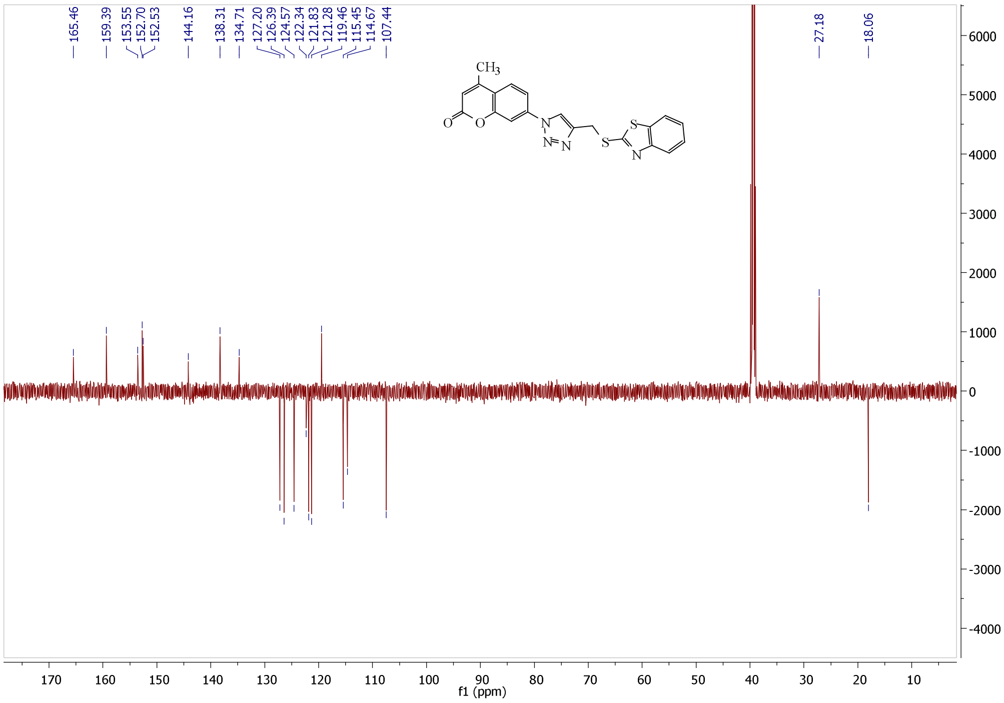

Figure S14. 7-{4-{[(1H-Benzo[*d*]imidazol-2-yl)thio]methyl}-1H-1,2,3-triazol-1-yl}-4-methyl-2H-chromen-2-one (**4b**)

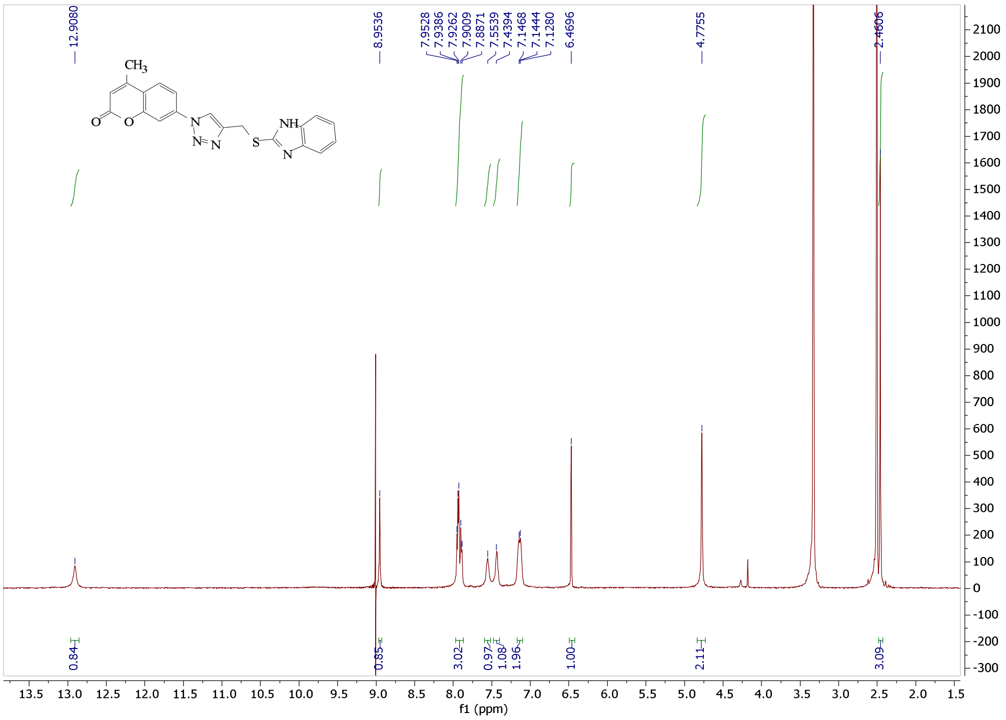

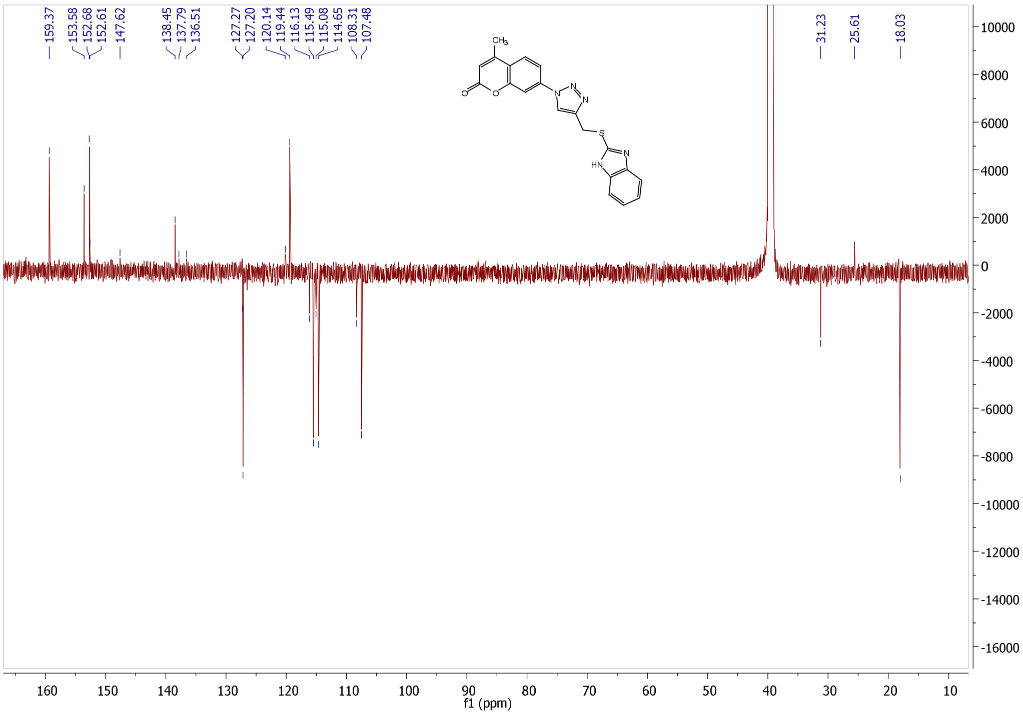

HRMS spectra

Figure S15. 4-Methyl-7-(4-phenyl-1H-1,2,3-triazol-1-yl)-2H-chromen-2-one (**2a**)

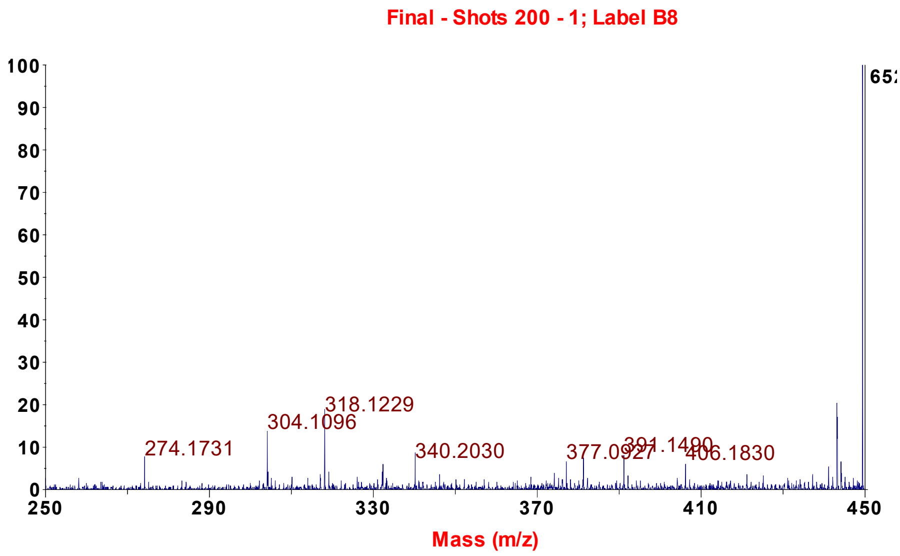

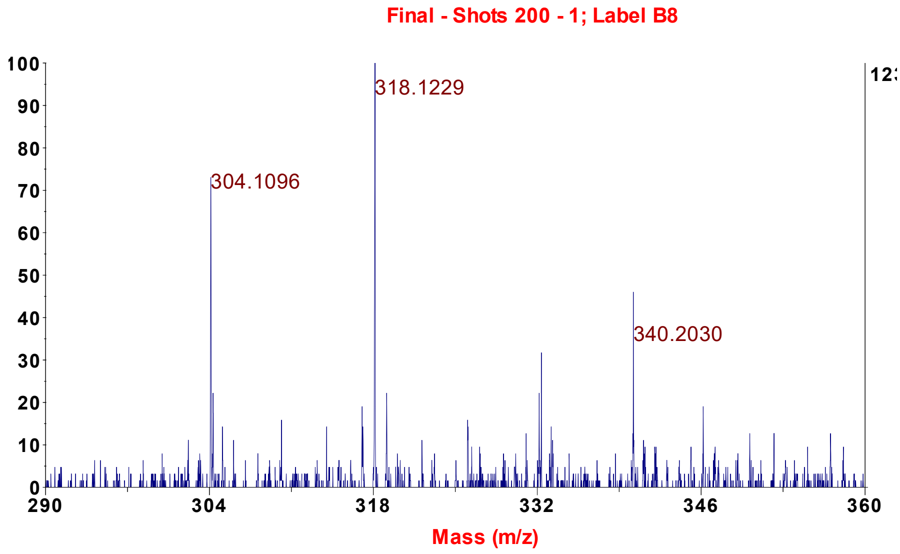

Figure S16. 7-[4-(4-Aminophenyl)-1H-1,2,3-triazol-1-yl]-4-methyl-2H-chromene-2-one (**2b**)

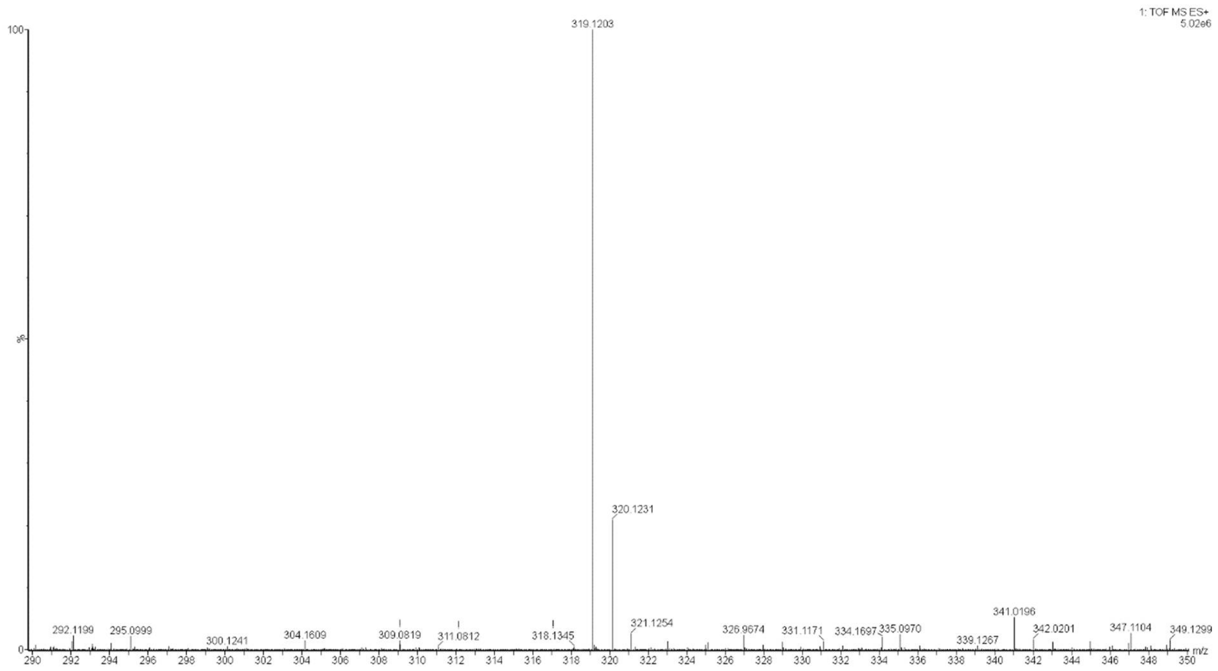

Figure S17. 7-[4-(4-(Dimethylamino)phenyl]-1H-1,2,3-triazol-1-yl)-4-methyl-2H-chromen-2-one (**2c**)

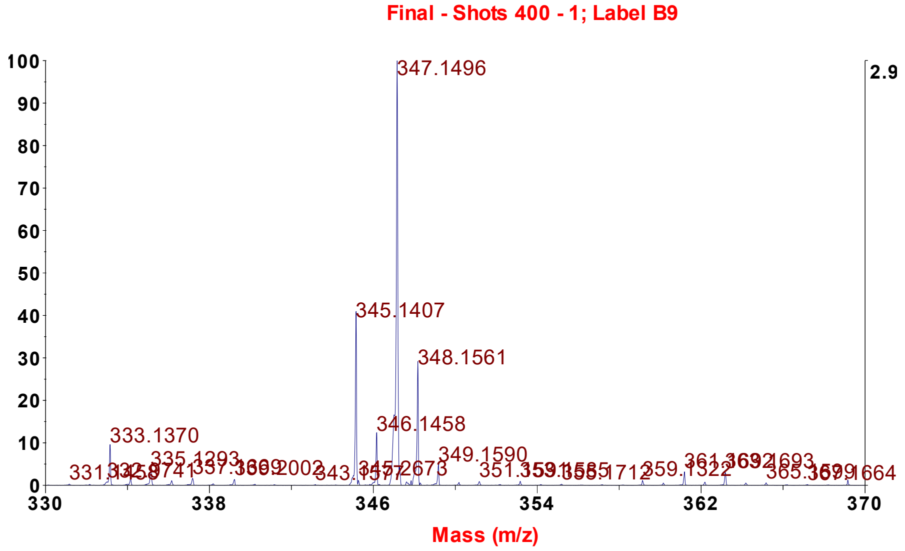

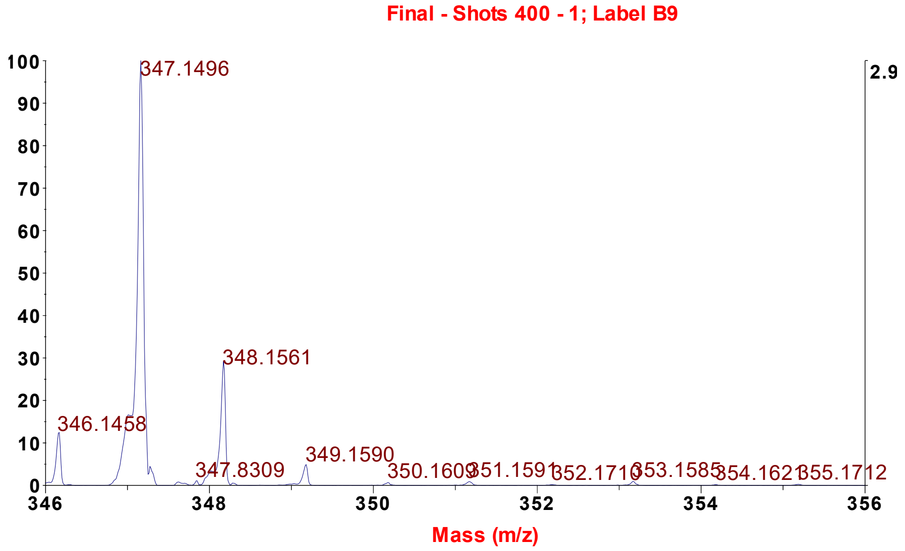

Figure S18. 4-Methyl-7-[4-(*p*-tolyl)-1H-1,2,3-triazol-1-yl]-2H-chromen-2-one (**2d**)

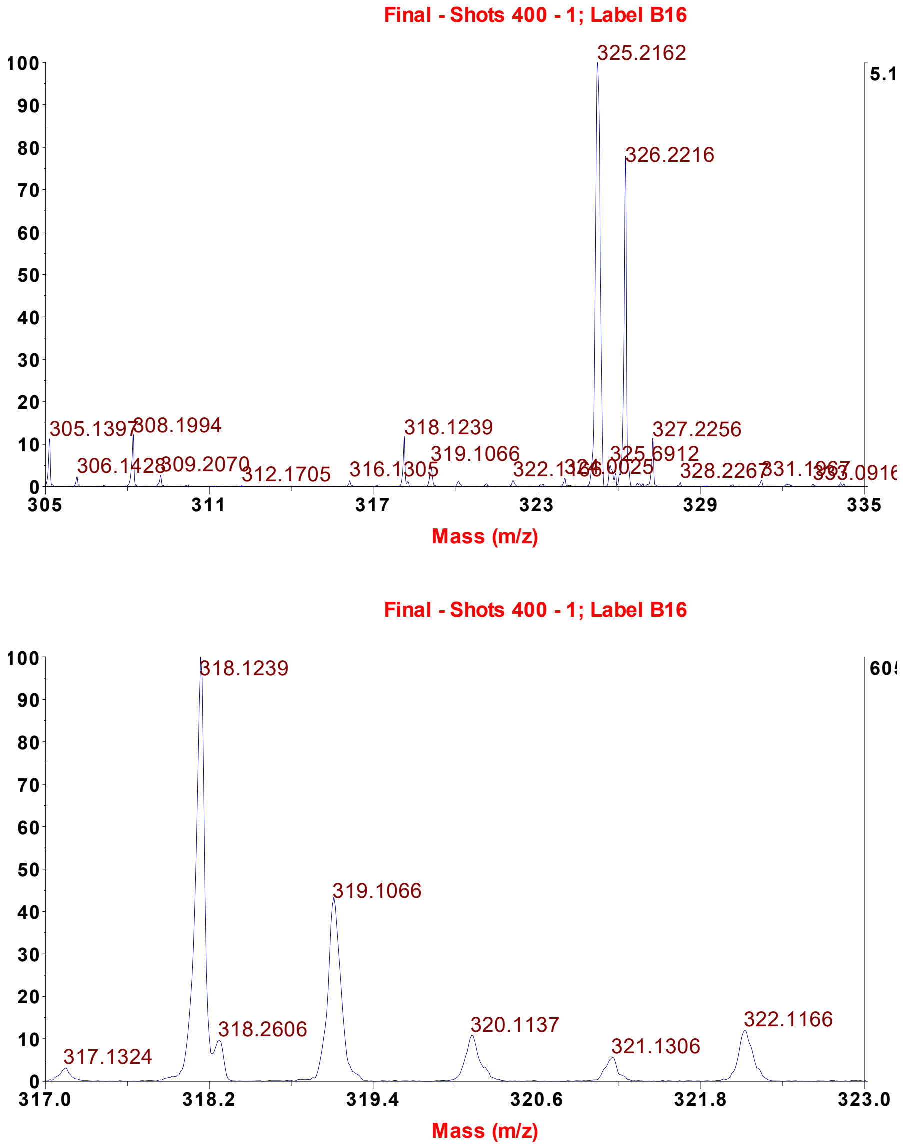

Figure S19. 7-[4-(4-Bromophenyl)-1H-1,2,3-triazol-1-yl]-4-methyl-2H-chromen-2-one (**2e**)

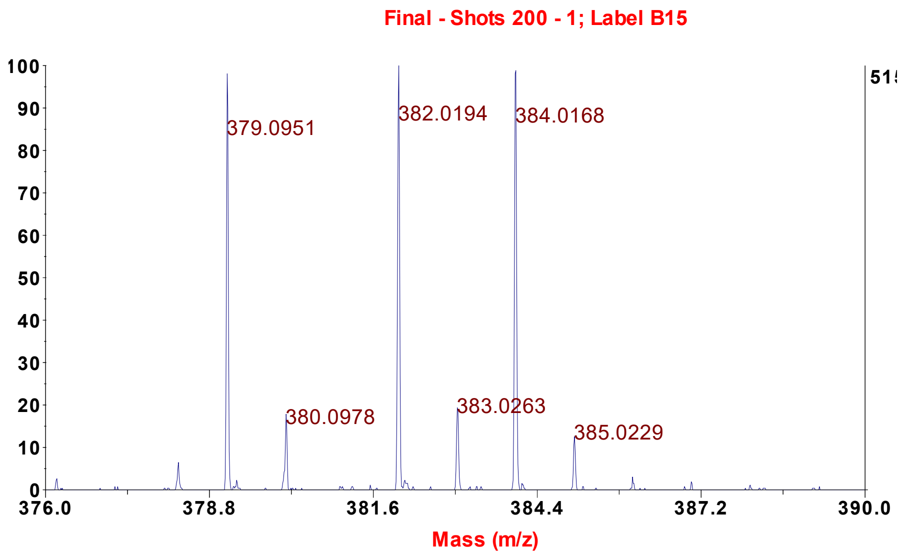

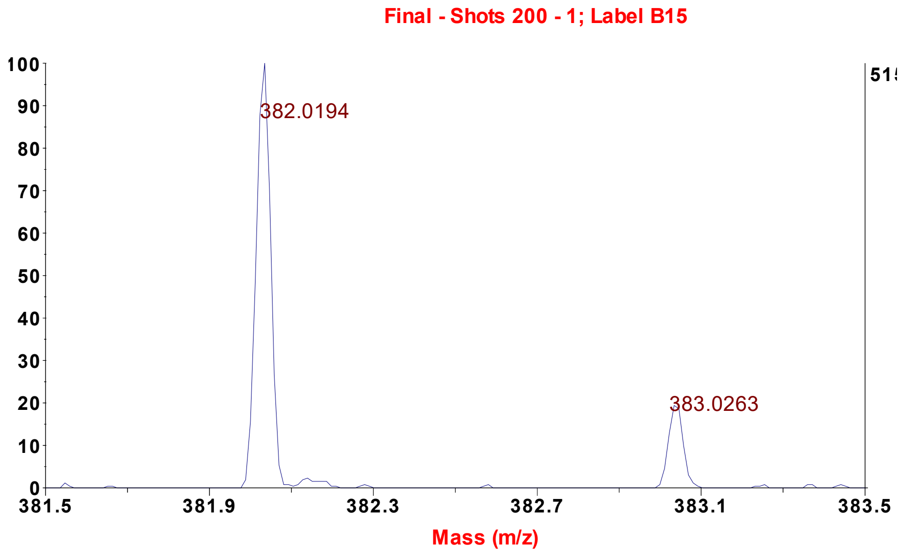

Figure S20. 7-[4-(4-Methoxyphenyl)-1H-1,2,3-triazol-1-yl]-4-methyl-2H-chromen-2-one (**2f**)

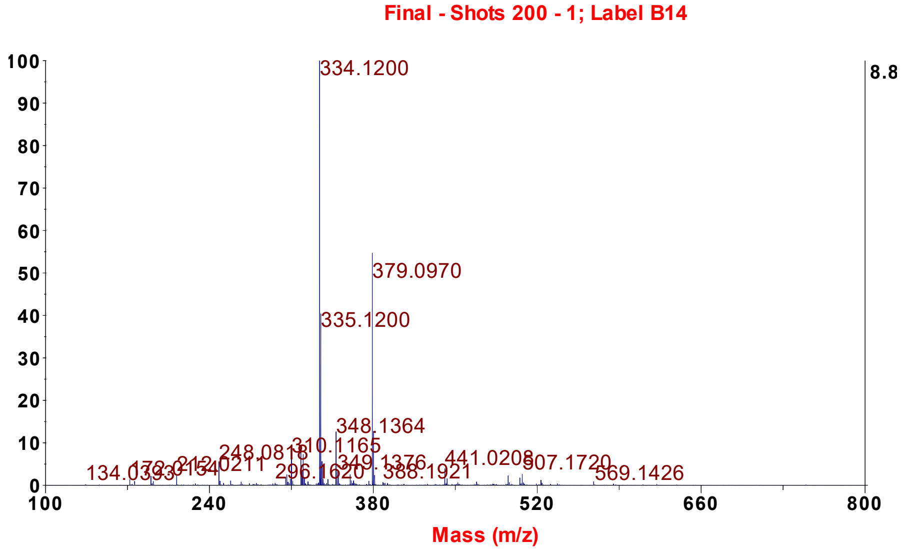

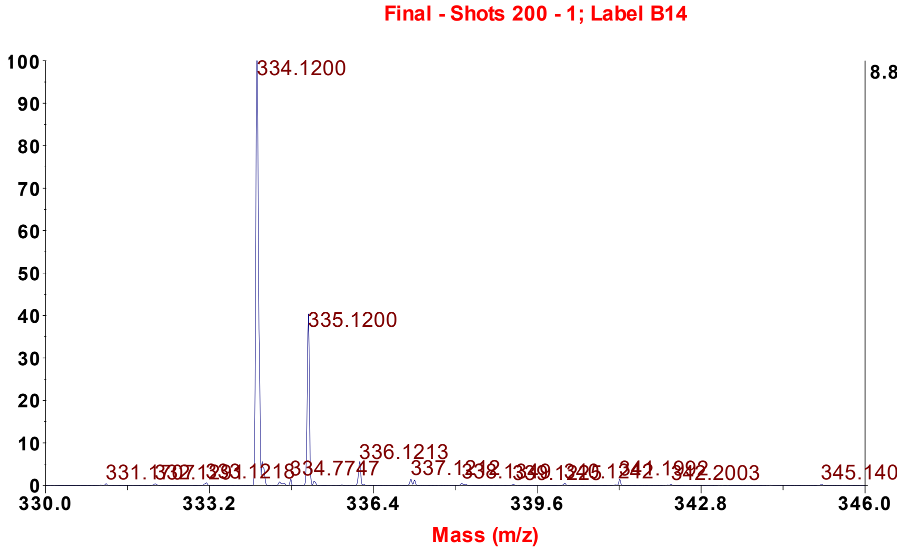

Figure S21. 4-[1-(4-Methyl-2-oxo-2H-chromen-7-yl]-1H-1,2,3-triazol-4-yl)benzaldehyde (**2g**)

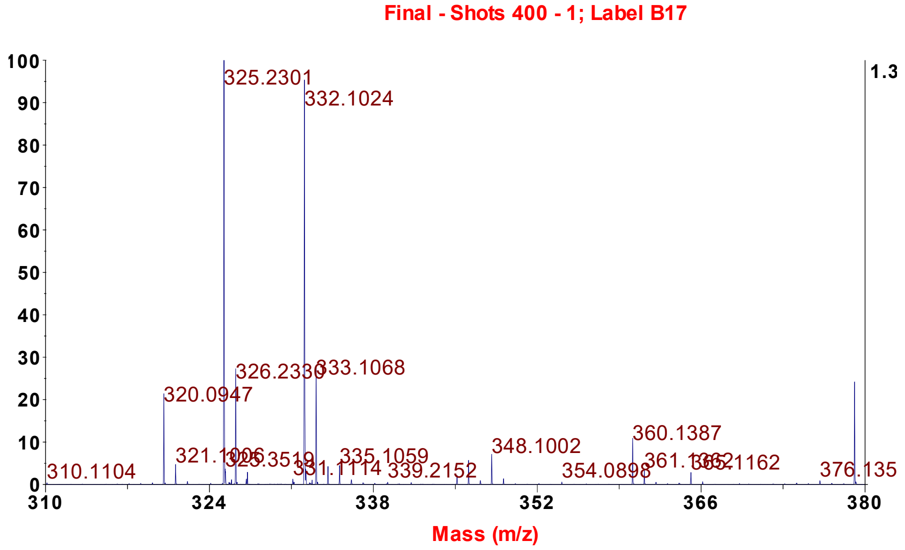

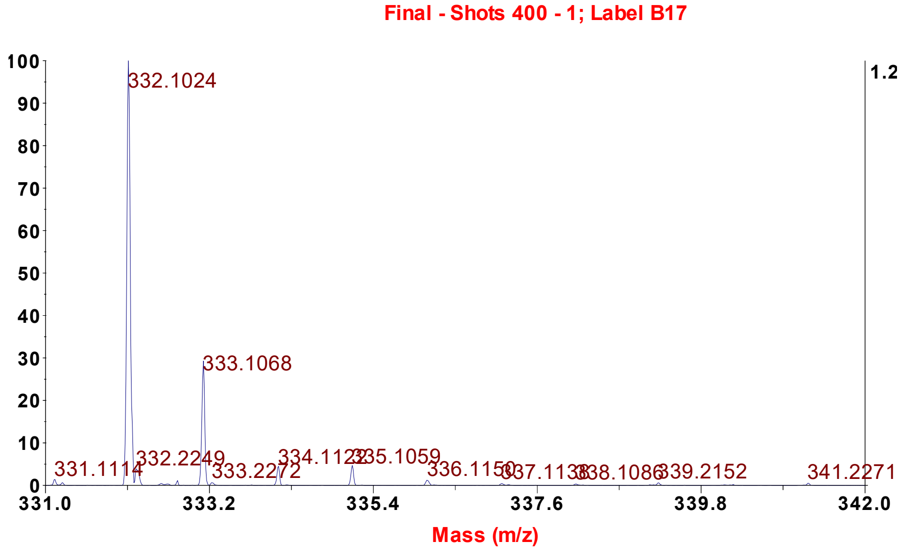

Figure S22. 7,7'-(4,4'-(1,3-Phenylene)bis(1H-1,2,3-triazole-4,1-diyl))bis(4-methyl-2H-chromen-2-one) (**2h**)

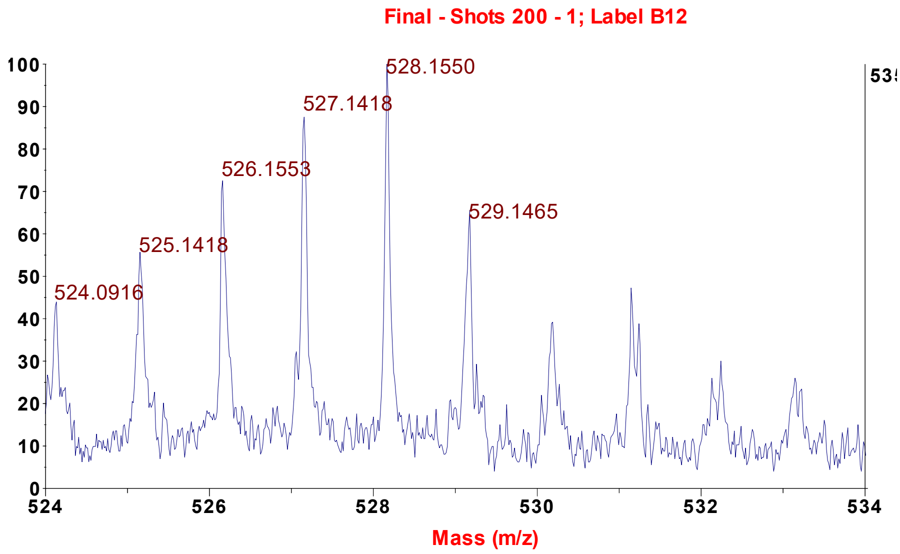

Figure S23. 7,7'-(4,4'-(1,4-Phenylene)bis(1H-1,2,3-triazole-4,1-diyl))bis(4-methyl-2H-chromen-2-one) (**2i**)

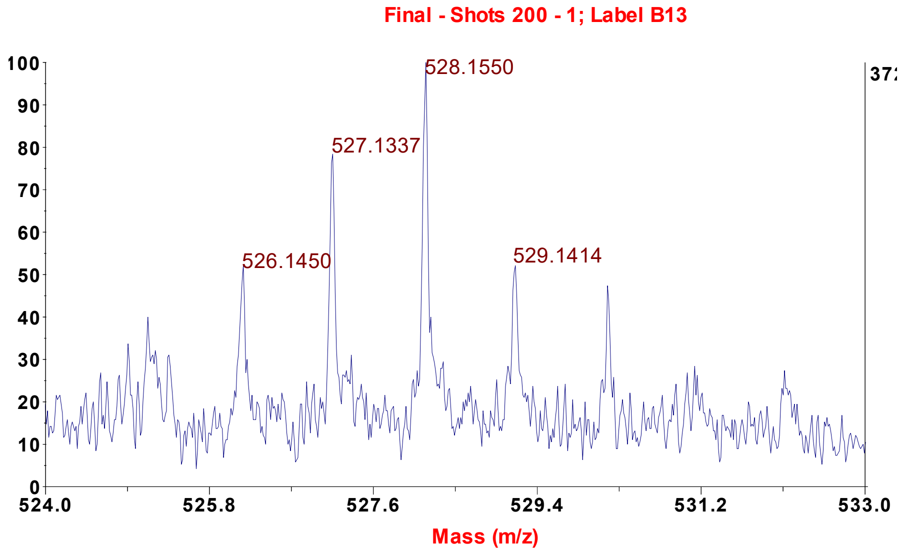

Figure S24. 7-{{4-[(Benzo[d]thiazol-2-yl)thio]methyl}-1H-1,2,3-triazol-1-yl}-4-methyl-2H-chromen-2-one (**4a**)

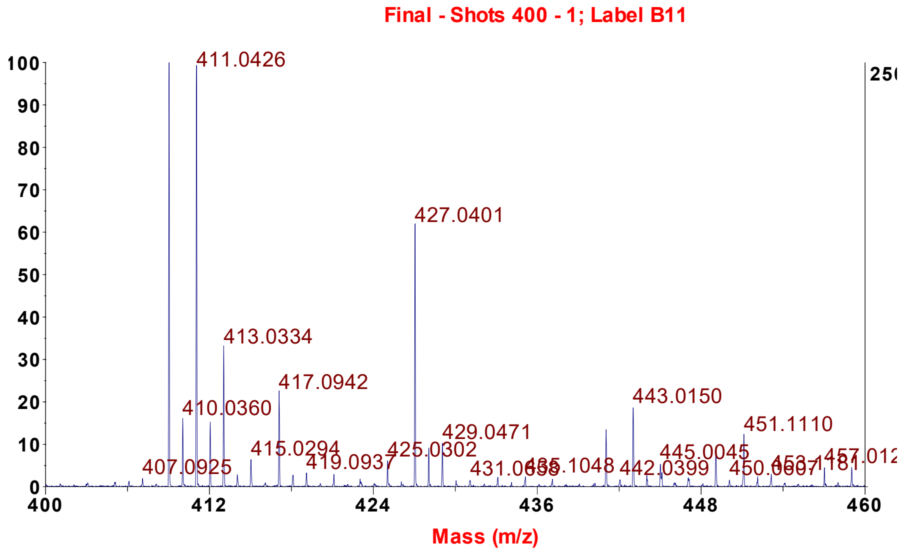

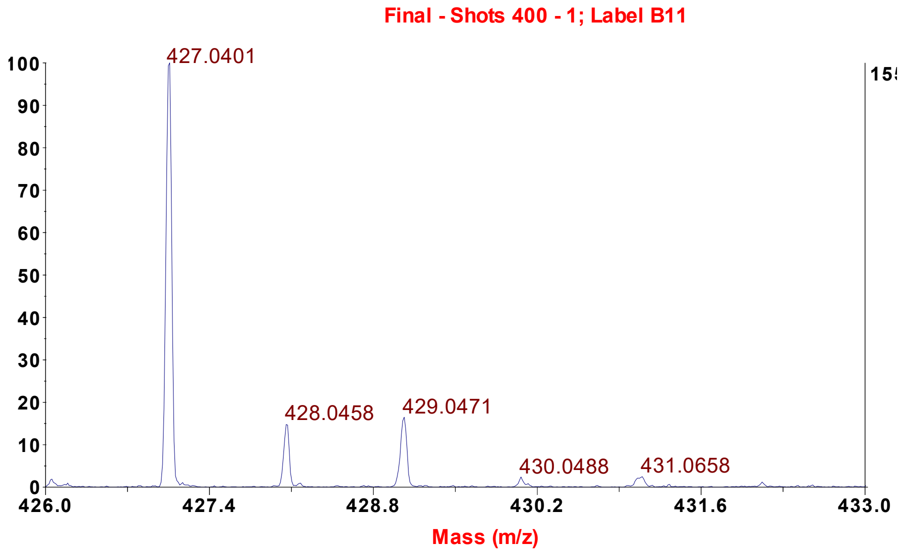

Figure S25. 7-{4-{[(1H-Benzo[d]imidazol-2-yl)thio]methyl}-1H-1,2,3-triazol-1-yl}-4-methyl-2H-chromen-2-one (**4b**)

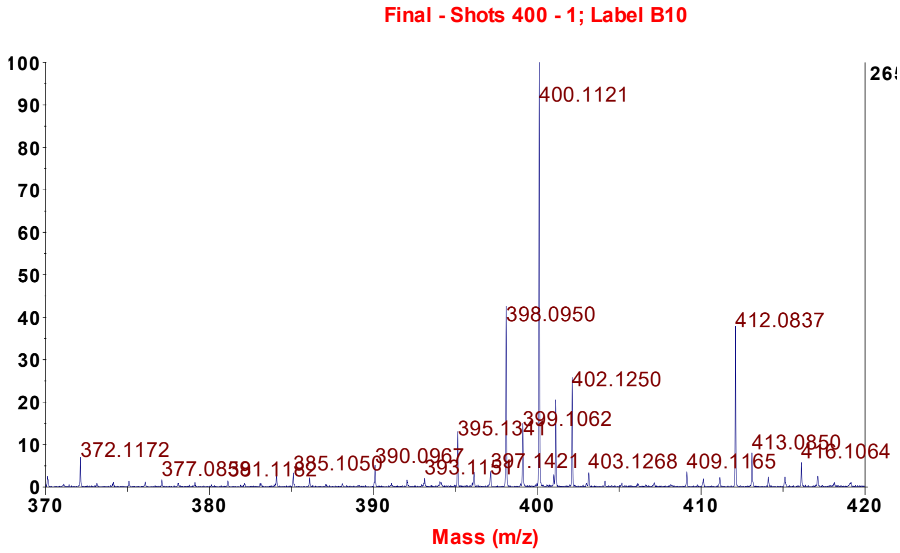

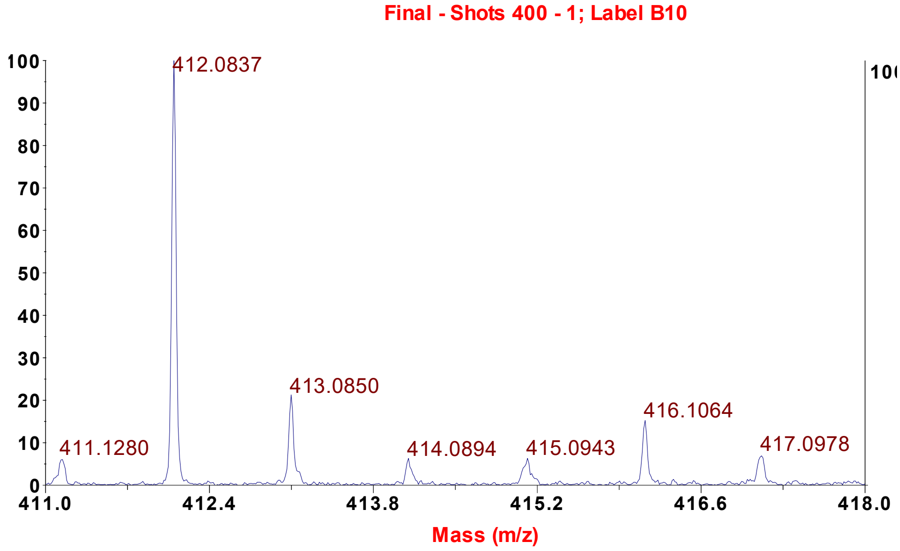

Supplement: Supplementary file 1 [file molecules-27-00637-s001.zip › molecules-1528391-supplementary.pdf]
